# Therapeutic Peptides Targeting PPI in Clinical Development: Overview, Mechanism of Action and Perspectives

**DOI:** 10.3389/fmolb.2021.697586

**Published:** 2021-06-14

**Authors:** Walter Cabri, Paolo Cantelmi, Dario Corbisiero, Tommaso Fantoni, Lucia Ferrazzano, Giulia Martelli, Alexia Mattellone, Alessandra Tolomelli

**Affiliations:** Department of Chemistry “Giacomo Ciamician”, Alma Mater Studiorum University of Bologna, Bologna, Italy

**Keywords:** therapeutics, clinical trials, PPI, peptides, pharmacokinetic, GLP-1, cancer, SPPS

## Abstract

Targeting protein-protein interactions (PPIs) has been recently recognized as an emerging therapeutic approach for several diseases. Up today, more than half a million PPI dysregulations have been found to be involved in pathological events. The dynamic nature of these processes and the involvement of large protein surfaces discouraged anyway the scientific community in considering them promising therapeutic targets. More recently peptide drugs received renewed attention since drug discovery has offered a broad range of structural diverse sequences, moving from traditionally endogenous peptides to sequences possessing improved pharmaceutical profiles. About 70 peptides are currently on the marked but several others are in clinical development. In this review we want to report the update on these novel APIs, focusing our attention on the molecules in clinical development, representing the direct consequence of the drug discovery process of the last 10 years. The comprehensive collection will be classified in function of the structural characteristics (native, analogous, heterologous) and on the basis of the therapeutic targets. The mechanism of interference on PPI will also be reported to offer useful information for novel peptide design.

## Introduction

In the progression of many pathological states, protein–protein interactions (PPIs) play the fundamental role of mediators of signal transmission and for this reason they represent optimal targets for drug discovery.

The size of the human interactome, the complex network of protein interactions, has been estimated to involve about 650,000 relevant contacts (Stumpf et al., 2008) that regulate all biological events and are responsible, when dysregulated, of a great number of pathological diseases. The dynamic nature of PPI and the large contact area that is often required between the partners (approximately 1,000–4,000 Å) have discouraged in the past to consider them as interesting targets. Small molecule drugs indeed are able to inhibit only interactions involving a binding area from 300 to 1,000 Å (Jones and Thornton, 1996) and PPI often lack of definite pockets. Usually protein association occurs between hydrophobic regions called “hot spots”, rich in amino acids that are able to form hydrogen bonds and π-interactions, and only a small number of crucial residues within the complete area are involved in determining binding affinity and specificity. Many hot spots core regions are associated with the presence of α-helix, β-sheet and β-turn protein secondary structure motifs and peptidomimetics targeting PPI have been tailored in the past years to mimic these ordered structures (Robertson and Spring, 2018; Mabonga and Kappo, 2020).

In this scenario, peptides have been discarded as potential lead compounds because they were affected by several issues, namely the random conformations of short sequences, the difficulties in synthesis and purification of longer ones and the sensitivity to endopeptidases, determining generally half-lives of few minutes (Fozgerau and Hoffmann, 2015). Moreover, susceptibility to proteolytic degradation made oral administration a challenge. The development of reliable techniques for peptide synthesis and purification gave access to pharmaceutical grade peptides, even with a high number of amino acids. In addition, molecular design can easily overcome the above-mentioned limits, thanks to the incorporation of non-native amino acids, the introduction of lipophilic side chains or cyclic sequences. For this reason, in the last two decades peptides have found renewed attention (Lau and Dunn, 2018) and up today about 70 therapeutic peptides have been approved and launched on the market. Among the reasons behind the development of new chemical entities (NCEs) in the polypeptide segment there is the commercial and therapeutic success of GLP-1 analogues, Liraglutide and Semaglutide, for the treatment of type 2 diabetes and obesity, that exceeded 7.6 B$ sales in 2020 (Novo Nordisk 2020 annual report, February 3, 2021). The 13 h half-life of liraglutide (Neumiller and Campbell, 2009) and the 7 days half-life of semaglutide (Hall et al., 2018), which is also orally available, have been at the basis of the success of these medicines. Several market studies forecasted a consistent global growth and success of the peptide segment, from 29 B$ sales in 2019 to 48 B$ in 2025 (excluding insulin), with an annual 10% increase (Mordor Intelligence, 2020).

We focused this review on the peptides under clinical trials in the last 3 years, that are the direct application of the increased PPI knowledge and of the synthetic design and tactics evolution during the last 10 years. Only therapeutic peptides with a length up to 40 aa, according to the FDA definition, are discussed (FDA - U.S. Food and Drug Administration, 2018). We strictly considered therapeutic peptides targeting PPI, excluding diagnostic, radiotherapeutics, vaccines and conjugated peptides with different chemical modalities (Blanco and Gardinier, 2020).

**FIGURE 1:**
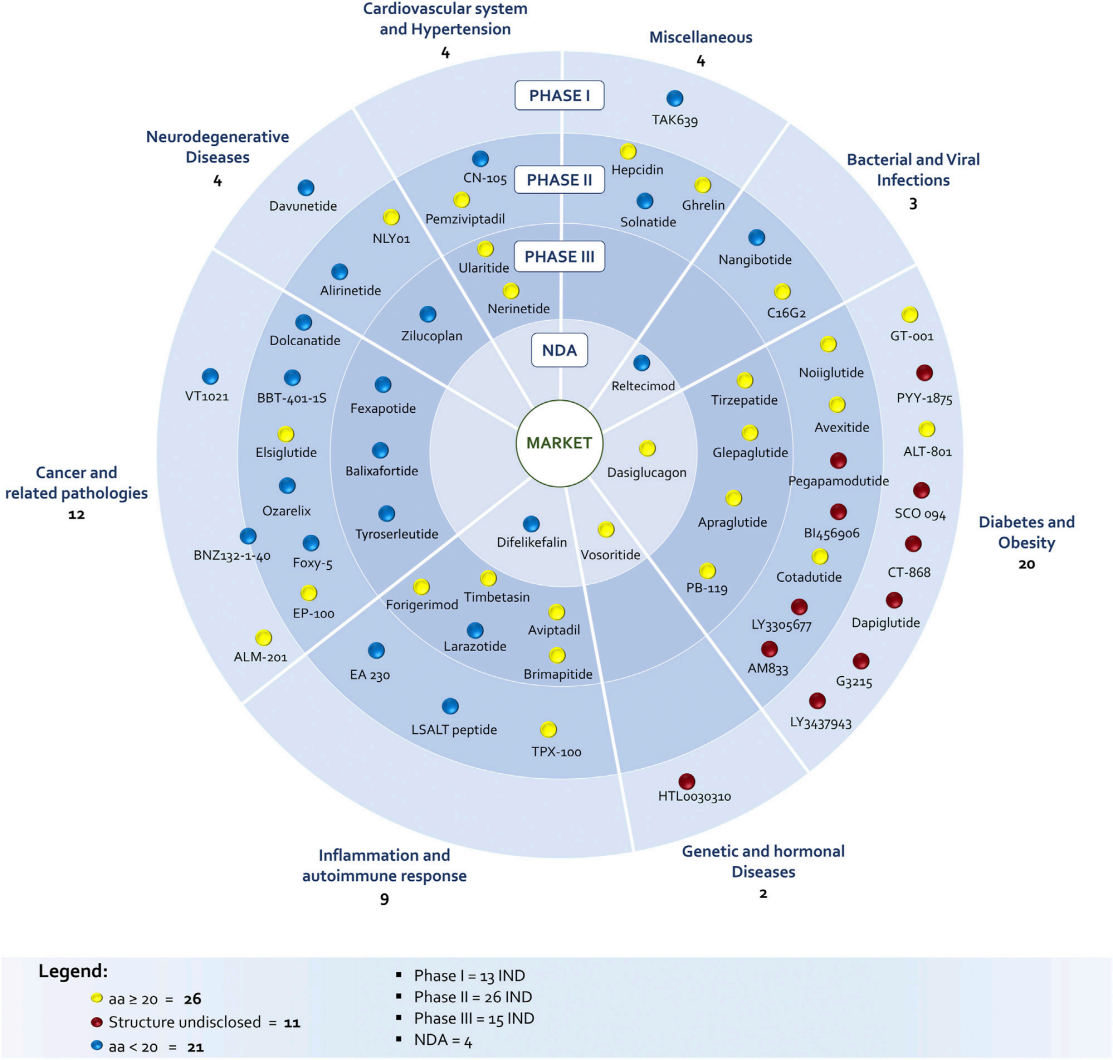
Clinical status of peptide with protein-protein interaction properties.

The identification of peptides in clinical phase was not a simple task. The list of peptides under development discussed in this survey is the combination and critical evaluation of information coming from Cortellis-Clarivate, clinical trials databases (United States and Europe) and from companies’ web sites, in order to really understand product status. In fact, during the drug discovery and development process few companies communicate NCEs elimination from the pipeline and most of the time the molecule simply disappears from official reports and websites. In addition, the same molecule can be identified with several codes and names due to product sales, co-development between companies, company mergers and acquisitions or definition of an international nonproprietary name. The list of discussed molecules includes 58 peptides in different clinical phases: 13 entered the Phase 1, 26 are in Phase 2, 15 are in Phase 3, while four are close to approval being the new drug application (NDA) already submitted to regulatory authorities (Figure 1). A striking difference in respect to the past is the increased number of amino acids, being the number of peptides with 20–40 residues prevailing on shorter peptides (26 *vs* 21) among the disclosed structures (47 of 58). However, this number should be even higher, considering that nine undisclosed structure are in diabetes and obesity segment, were peptides are typically longer than 20 AA.

With the only exception of two peptides, GT001 and Elsiglutide, that are produced through recombinant technology, all the other compounds with disclosed sequence are synthetically produced, commonly via solid phase approach (SPPS). In this context, many groups worldwide are targeting green innovative techniques (Lawrenson et al., 2017; Ferrazzano et al., 2019; Nuijens et al., 2019; Martin P. et al., 2020). As for the pathologies, diabetes, obesity, cancer and related pathologies are the major areas of interest.

**FIGURE 2:**
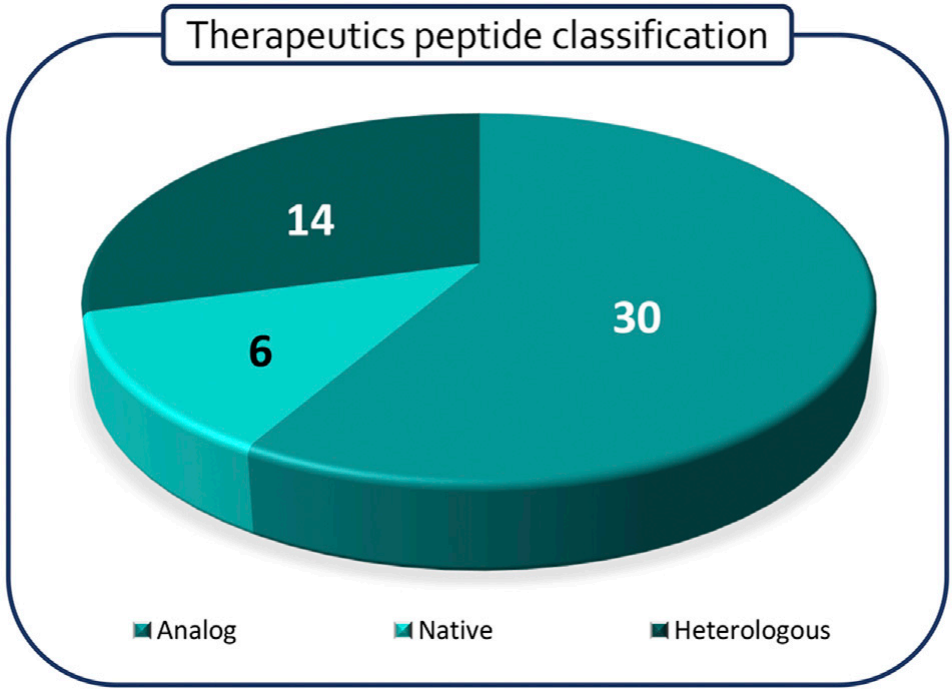
Classification of discussed peptides according to Lau/Dunn classification.

**FIGURE 3:**
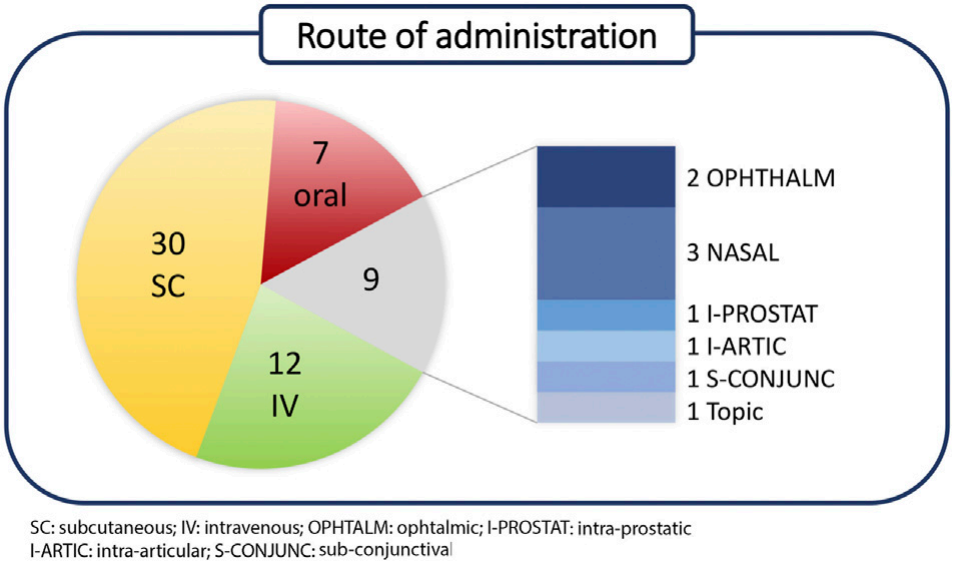
Classification of discussed peptides on the basis of RoA.

Polypeptides have been classified according to Lau/Dunn definition (Lau and Dunn, 2018): **native** peptides have the same sequence of endogenous ligand, while **analogous** are products where the natural sequence has been modified in order to achieve a better pharmacological profile. **Heterologous** peptides have been discovered independently respect to the natural ligand and are based on a more classical medicinal chemistry approach (Figure 2). It is worth to notice that native peptides and their analogues represent more than 70% of the PPI targeting peptides in clinical trials, being in particular, the analogue ones accounting for 60% of the pipeline. The peptides have been discussed on the basis of the target pathology and, besides the clinical trial stage, the route of administration (RoA), as a symptom of peptide pharmacokinetic properties, has been considered (Figure 3).

## Cancer and Related Pathologies

The role of PPI in the development of tumors is strictly connected to protein-mediated signalling processes, able to activate several biological networks related to tumorigenesis, progression, invasion, and metastasis. For this reason, introducing perturbations in PPI represents an efficient strategy for interrupting cancer-related phenomena. Looking at different tumor phenotypes, specific proteins undergo modifications in their interaction pattern when they become part of carcinogenic phenomenology, with important effects on a patient’ state of health. Depending on the tumor localization, it is possible to target different candidate proteins, generally transmembrane receptors or releasing hormone receptors, with theranostic performances useful in understanding cancer progression and improving treatment efficacy (Gulfidan et al., 2020).

Concerning the role of transmembrane receptors in cancer development, **Dolcanatide** was designed for the treatment of inflammatory bowel disease and functional gastrointestinal disorders to prevent colon cancer. In fact, cells belonging to intestinal epithelium are responsible for controlling toxic pathogens’ efflux through the digestive apparatus and transport of nutrients, fluids, and bacterial flora (Rappaport and Waldman, 2018). To maintain homeostatic control of these processes the intestinal epithelial receptor, guanylate cyclase C (GUCY2C), and its cyclic nucleotide second messenger, cyclic guanosine monophosphate (cGMP), play a critical role. This heterodimeric transmembrane receptor can be activated by endogenous extracellular peptide ligands or other signalling agents, such as Ca^2+^ and nitric oxide (Boulete et al., 2018). Known ligands for GUCY2C receptor are the bacterial heat-stable enterotoxin, ST (cause of traveller’s diarrhea), and the endogenous peptides, uroguanylin and guanylin. Dolcanatide was designed to be an analogue of uroguanylin with enhanced resistance to standard digestive breakdown by proteases in the intestine as consequence of the presence of a disulfide bridge. The peptide sequence differs from uroguanylin for the substitution of Asp with Glu at the 3-position at the N-terminus, for greater binding affinity, and for L-Asn1 and L-Leu16 replaced by D-Asn1 and D-Leu16 at the N- and C-termini, respectively, which are thought to provide enhanced biostability (Shailubhai et al., 2015). Dolcanatide is supposed to bind GUCY2C similarly to uroguanylin and the other known endogenous ligands, via the NH_2-_terminal β-hairpin, by providing the third strand of a small triple-stranded antiparallel β-sheet (Lauber et al., 2003).

Similarly, **BBT-401** (structure undisclosed) is an orally available Pellino-1 protein-protein interaction inhibitor for the treatment of patients affected by ulcerative colitis. This lipidated tetrapeptide binds Pellino-1 protein, interfering in its signalling cascade. BBT-401 efficiently inhibited the lipopolysaccharide-induced activation of the toll-like receptor proinflammatory signalling pathway, reducing proinflammatory cytokine expression as well. Studies on animal models showed that BBT-401 administration in colitis significantly improved symptoms and histopathological parameters associated to the disorder, and direct colon administration enhances considerably the therapeutic efficacy (Lee et al., 2019).

**FIGURE 4:**
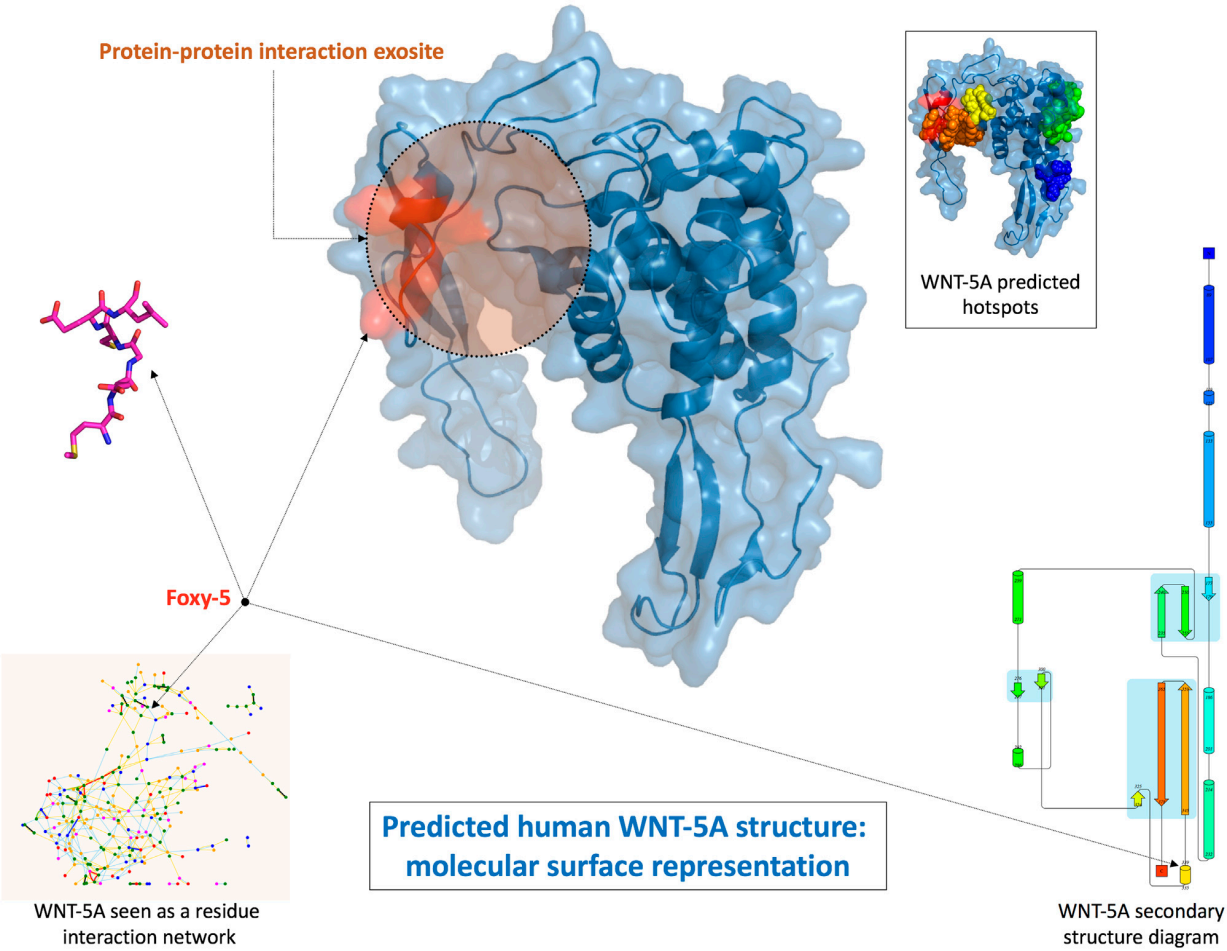
Structural prediction of human WNT-5A and Foxy-5 conformation (Villoutreix, 2017).

Interfering on surface receptors functions seems to be particularly relevant for solid and metastatic tumor progression. **Foxy-5** is a formylated WNT5A-derived six amino acid peptide, recently used in a Phase 1 trial in patients with metastatic breast, colon and prostate cancer and in a Phase 2 trial for early stage colon cancer. Its model protein, WNT5A, is a member of the Wnt protein family, which plays important roles in several physiological phenomena, like organ development, tissue orientation, cell polarity and migration. Its dysregulation has been associated with progression of various diseases as a consequence of its tumor-suppressive function in colon cancer, neuroblastoma, breast carcinomas, and leukemia (Säfholm et al., 2008; Canesin et al., 2017). In this contest, Foxy-5 has been characterized as a WNT5A-mimicking peptide, since it acts by triggering cytosolic free calcium signalling and by impairing migration and invasion of epithelial cancer cells. In silico prediction suggests that Foxy-5 could adopt in solution a short loop and alpha helical structure, a motif quite common in protein-protein interactions. In the structure of the full-length native protein, this segment is solvent exposed and could be involved in macromolecular interactions. Foxy-5 seems to be part of an exosite, expected to be critical for the interactions of WNT-5A with receptors and be part of a dimerization site (Figure 4, Villoutreix, 2017).

**VT1021** is a cyclic pentapeptide developed by Vigeo Therapeutics whose structure is still undisclosed. It acts as a potent inducer of thrombospondin-1 (Tsp-1) expression in the tumor microenvironment (TME), which is then totally reprogrammed to reduce its immune-suppressive and tumor-promoting propensity, promoting instead the activation of the adaptive immune system and its tumor-inhibiting inclination (Harb et al., 2019). VT-1021 is at Phase 1 of clinical trials for the treatment of solid tumor since, in preclinical animal models, it showed robust anti-tumor activities on ovarian, pancreatic and breast cancer, with complete tumor regression and reprogramming of the immune TME.

**Balixafortide** is another good example of a potent and selective antagonist of the chemokine receptor CXCR4, which belongs to the transmembrane receptor family of G-coupled protein receptors (GPCR). This protein is largely expressed in several tumors type, including expression by cells of immune system in carcinogenic tissues. The role of CXCR4 in metastatic phenomena is particularly relevant, since this protein allows cancer cells to migrate to other sites where its natural ligand CXCL12 is expressed, like in the bone marrow of patients affected by breast cancer (Zimmermann et al., 2019). This evidence justifies the application of Balixafortide, in combination with eribulin, in Phase 3 trial for metastatic breast cancer and in a more recent Phase 1 trial for a dose-escalation study (Kaufmann et al., 2020).

In cancer progression and tumorigenesis, interleukin dysregulation has an important role, and its upregulation seems to be associated with many tumors. For this reason, controlling its functions is advantageous for blocking cancer development. **BNZ132-1-40** is a 19-mer pegylated peptide belonging to the anticytokine peptide family, active selectively on IL-15, IL-2 and IL-9, which drives T-cell mediated diseases including T-cell large granular lymphocyte leukemia (T-LGLL) and HTLV-1 driven adult T-cell leukemia (ATL) (Massoud et al., 2015; Wang et al., 2019). The peptide structure with helical conformation is responsible for the direct binding to γc receptor, present in all the above-mentioned cytokines which are fundamental for leukemia survival and lack of strong active therapies. Furthermore, the presence of a PEG-chain was required to increase the peptide half-life. The selectivity of BNZ132-1-40 for Tregs, CD8^+^ T and NK cells was confirmed by the absence of adverse effects on other immune cells and the tolerance showed by healthy subjects reported in a recent Phase 1 clinical trial (Nata et al., 2015).

**ALM-201,** that is a 23-mer peptide drug candidate, is a synthetic derivative of AD-01, a 24-mer peptide containing the sequence 34-58 of FK506-binding protein like (FKBPL). This protein, that showed prognostic potential in breast cancer, can be classified both as extracellular and intracellular protein: in the first case, it acts as a secreted anti-angiogenic protein targeting cell surface receptor CD44, upregulated in carcinogenic scenario, while intracellularly FKBPL shows a prognostic activity for breast cancer survival. As a consequence, FKBPL is involved in many cellular processes like cell cycle progression, signalling and differentiation (Annett et al., 2020). ALM201, like other FKBPL derivatives, inhibits breast cancer metastasis through Notch signalling, showing excellent safety profile in Phase 1 clinical trials, when applied for the treatment of ovarian cancer and solid tumor (McClements et al., 2019).

In some cases, the complexity of cancer physiopathology does not allow to clearly identify a single target protein to control cancer progression, even if efficient drug treatments can be applied. As an example, **Fexapotide triflutate** is a 17-mer peptide in clinical development for prostate cancer and hyperplasia. Even if its mechanism of action remains partially undisclosed, it is known that Fexapotide works stimulating several protein pathways, like caspase, tumor necrosis factor and BCL pathways in prostate glandular epithelial cells. It induces selective cell membrane permeability, mitochondrial metabolic arrest and reduces RNA, DNA lysis and aggregation, with subsequent cell fragmentation and loss, causing decompression of the urethral lumen (Shore et al., 2018). Studies on animal models revealed that Fexapotide direct injection in injured tissues, like bladder, urethra, rectum, periprostatic tissue, leaves unaffected adjacent tissues. For all these evidences, it reached Phase 3 in clinical trials for urinary-related pathologies.

Similarly, **Tyroserleutide** (YSL) is a tripeptide compound active on primary hepatocellular carcinoma, in Phase 3 of clinical trials. This short peptide, which showed very high activity and few side-effects, was originally purified from the hydrolysates of pig’s spleen, but now obtained through chemical synthesis. To carry out its antitumor activity, YSL involves the second messenger Ca^2+^, which is able to modulate cell function by Ca^2+^/calmodulin (CaM) pathway. In addition, CaM associates with phosphatidylinositol three kinase (PI3K), enhancing its activity and promoting upregulated cell proliferation (Zhao et al., 2008).

For the treatment of cancer types associated with the functioning of endocrine systems or for controlling side-effects related to other chemotherapeutic treatments, hormone receptors release appears a good targeting strategy to control and stop tumor escalation. **Elsiglutide**, a selective glucagon-like peptide of type 2 (GLP-2) derivative, was developed as adjuvant to chemotherapy to reduce induced diarrhoea. This new synthetic versions of GLP-2 and elsiglutide show longer half-life, reducing the frequency of administration. (Mayo et al., 2020). The peptide completed successfully a Phase 2 clinical trial.

**Ozarelix** is a fourth generation Luteinizing Hormone-Releasing Hormone (LHRH) antagonist acting on Gonadotropin-releasing hormone (GnRH) receptor, which controls the secretion of luteinizing and follicle-stimulating hormone from the anterior pituitary gland. As other LHRH analogues, it is a 10-mer peptide with several D-amino acids and bulky residues (in particular in position 6) to enhance stability against proteolysis. Gonadotropin-releasing hormone is a decapeptide (EHWSYGLRPG), displaying an *N*-terminal trimeric fragment responsible for agonist activity and a C-terminal trimer necessary for affinity to the receptor. Since the predominant bioactive conformer consists of a β-turn, where the two terminals are close together, analogues have been generally designed by replacing the central glycine in position 6 with residues able to stabilize the requested turn (Flanagan and Manilall, 2017). At first, Ozarelix was designed to stimulate secretion of gonadotropins, but continuous evidence demonstrates a decrease of pituitary hormone secretion, as a consequence of gonadotrophic cells desensitization and down-regulation of pituitary receptors (Festuccia et al., 2010). In fact, as GnRH antagonists, Ozarelix induces reduction in gonadotropin levels by inhibition of their secretion from the anterior pituitary gland. Ozarelix completed Phase 2 of clinical trials for the treatment of prostate cancer, showing higher solubility, definitive suppression of testosterone and any risk of clinical flare if compared to other antagonists (Schneider et al., 2010).

**EP-100** is a synthetic LHRH natural ligand derivative made up 28 amino acid, 18 of which belong to the cationic α-helical lytic peptide (CLIP-71). Luteinizing-hormone-releasing hormone (LHRH) receptors overexpression has been found in several tumor types, like prostate, breast, ovarian, endometrial, pancreatic, bladder, colorectal, melanoma and non-Hodgkin lymphoma. For this reason, it has been selected as privileged target for treatment of solid and ovarian tumors, where EP-100 is in Phase 1 and 2 of clinical trials, respectively. LHRH sequence (HWSYGLRP) is responsible for the delivery of the lytic peptide to cancer cells via specific binding to the receptor LHRH on cell-surface receptors. The mode of action of EP-100 is not completely known. In fact, the negatively charged outer membrane of cancer cells determines the high selectivity of this peptide for carcinogenic tissues: after binding of LHRH targeting sequence, the positively charged portion of EP-100 interacts with the outer membrane of cancer cells in a disruptive manner, causing cell lysis and death (Curtis et al., 2014; Kim et al., 2020).

## Inflammation and Autoimmune Response

Inflammation and autoimmune responses are essentials body’s defence mechanisms. They are the processes by which the immune system recognizes and removes harmful and foreign stimuli and begins the healing processes (Pahwa et al., 2021). However, when an adaptive immune response develops against self-antigens or there is an upregulated recruitment of pro-inflammatory molecules, it is usually impossible for immune effector mechanisms to completely eliminate the antigen/pro-inflammatory molecules, and therefore a sustained response occurs (Janeway et al., 2001). In this perspective, peptide-based treatments able to interfere with the dysregulated body’s defence were developed.

**Forigerimod** (ImmuPharma, 2019) is a synthetic 21-mer linear peptide, phosphorylated in correspondence of Ser11 position, produced by ImmuPharma and designed from the small nuclear ribonucleoprotein U1-70 (Zimmer et al., 2013). As suggested by its commercial name, Lupuzor™ is employed for the treatment of Systemic Lupus Erythematosus (SLE), a chronic, life-threatening autoimmune, inflammatory disease affecting multiple organs such as skin, joints, kidneys, blood cells, brain, heart and lungs. Due to the high relevance of this pathology, Lupuzor™ received fast-track designation from the FDA, which expedites the drug’s approval process (ImmuPharma, 2021). Currently, the mechanism of action of Lupuzor™ has not been fully elucidated; however, several studies have shown that it displays tolerogenic and immunomodulatory effects leading to the inhibition of T cells’ reactivity in presence of endogenous peptides.

On the other hand, for the treatment of Systemic Inflammatory Response Syndrome (SIRS) the intravenous infusion of **EA-230,** a synthetic 4-mer linear peptide produced by Exponential Biotherapies, is currently under investigation (Phase 2). The sequence was designed on the basis of the β-loop contained in the β subunit of the endogenous peptide human Chorionic Gonadotropin (hCG) (van den Berg et al., 2011). The SIRS pathology is caused by an upstream of pro-inflammatory agents (such as IL-6) and this dysregulated inflammatory response often results in tissue damage, failure of one or more organ systems, and high mortality. Even though the mechanism of action EA-230 is still unclear, biological evidence have demonstrated how changes of the hormonal milieu play a central role in the anti-inflammatory response (van Groenendael et al., 2019).

Another peptide employed to cure inflammation diseases is **Difelikefalin** acetate, a synthetic 5-mer linear D-peptide produced by Cara Therapeutics and designed starting from the sequence of the endogenous peptide Dinorphin A. As such, Difelikefalin acetate is an agonist of κ-opioid receptor (KOR) for the treatment of Chronic kidney disease-associated pruritus (CKD-aP), or uremic pruritus, and it has completed Phase 3 clinical trials in 2020 (Fishbane et al., 2020). CKD-aP is a severely distressing condition that occurs in more than 60% of patients undergoing dialysis and, even if the pathogenesis is still incompletely understood, opioid imbalance has been identified as a possible reason of the arise of this pathology (Lipman and Yosipovitch, 2020). Due to the ubiquitous nature of KOR (in peripheral and central nervous system), Difelikefalin acetate was designed as a selective agonist, avoiding the penetration into the CNS that aims to provide the benefits of minimizing itch through activation of the anti-pruritic KOR system without causing CNS side effects.

Moving to inflammation affecting the respiratory system, it is possible to mention the **LSALT peptide**, a synthetic 16-mer linear peptide developed by Arch Biopartners and still ongoing in Phase 2 clinical trials (Arch Biopartners, 2021) for the treatment of Acute Respiratory Distress Syndrome (ARDS). A hallmark feature of this inflammation is the recruitment of neutrophils from the bloodstream into inflamed tissue (Choudhury et al., 2019). In this context, dipeptidase-1 DPEP1, an anchored membrane protein, has been identified as the major adhesion receptor on the lung and liver endothelium for neutrophil sequestration, independently from its enzymatic activity. As a consequence, the inappropriate recruitment of neutrophils to various organs contributes to multi-organ dysfunction (such as pulmonary dysfunction). In order to prevent this healthy disfunctions, several experimental models have highlighted that LSALT peptide binds to DPEP-1 not inhibiting its enzymatic activity but preventing the neutrophils sequestration and the inflammation response.

Remaining within the ARDS treatment, **Avipdtadil** is a synthetic 28-mer linear peptide developed by NeuroRX and Relief Therapeutics Holding, designed starting from endogenous Vasoactive Intestinal Peptide (VIP) structure. This endogenous protein is involved in the development of pulmonary hypertension (PH), a progressive vascular disease caused by vasoconstriction and structural remodeling of arterioles, leading to dyspnea, fatigue, cough, chest pain, palpitations, peripheral edema, syncope, right heart failure, and death. Moreover, without treatment, PH can evolve in ARDS halving the survival rate within 5 years (Hu et al., 2015). As VIP analogue, Aviptadil through binding to G-protein coupled receptors (VPAC1, VPAC2 and PAC1) induces pulmonary vasodilation and shows anti-inflammatory properties. In fact, Aviptadil aerosol is still ongoing in Phase 2/3 for the treatment of ARDS leading to a small and temporary but significant selective pulmonary vasodilation, an to an improved stroke volume and mixed venous oxygen saturation (Leuchte et al., 2008).

**Larazotide**, a 9 m Biopharma’s investigation drug (9 Meters Biopharma Inc., 2021), is the only therapeutic candidate for celiac disease adjuvant therapy, advanced to a Phase 3 clinical trial, that minimizes symptoms in tandem with gluten free diet. This octapeptide derives from the *Zonula Occludens* toxin secreted by *Vibrio cholerae* and it is an antagonist of zonulin, the only known physiological modulator of the intercellular tight junctions and a key player in regulation of the mucosal immune response in small intestine (Heickman et al., 2020).

Moving to more specific areas of the body, the treatment of the hearing and ocular inflammations is particularly interesting. **Brimapitide** is a synthetic dextrogyre 31-mer linear peptide developed by Auris Medical and designed from a combination of a 20-mer sequence of protein islet-brain 1 (IB-1) and 10-mer trans-activator of transcription sequence (TAT) of the HIVTAT protein, which allows its intracellular penetration. Its similarity with IB-1 makes this peptide a selective inhibitor of c-Jun N-terminal Kinase (JNK), a ubiquitous intracellular enzyme. (Chiquet et al., 2017). Its inhibition prevents formation of transcription complexes and further progress along the apoptotic pathway or activation of genes, which are encoding inflammatory molecules (such as cytokines). For this reason, Brimapitide is under investigation (Phase 3 clinical trials) for the treatment of acute sensorineural hearing loss (ASNHL) and ocular inflammation as biocompatible intratympanic hyaluronic acid gel and ophthalmic solution, respectively (Suckfuell et al., 2014).

Regarding the treatment of ocular inflammation, it is worth to mention **Timbetasin**, a synthetic 43-mer linear peptide produced by RegeneRX Biopharmaceuticals which behaves as analogue of the endogenous peptide Thymosin β4 from which it differs in the N-terminus acetylation. As such, Timbetasin is involved in the treatment of moderate to severe dry eye disease, thanks to its anti-inflammatory effect. Dry eye is a chronic ocular surface disease, causing visual morbidity that affects the quality of life, and it’s associated with an increase in the levels of inflammatory cytokines in both the conjunctiva and tears (Sosne et al., 2015). Generally, endogenous Thymosin β4 promotes wound repair and regeneration in the skin, eye, heart, and nervous system in various animal models; moreover, it is known to modulate the expression of multiple signalling molecules, playing a role in the downregulation of transcription factors for inflammatory chemokines, cytokines, and metalloproteinases (Sosne and Ousler, 2015). For this reason, the topical supplementary administration as eye drop solution of Timbetasin is currently under investigation (Phase 3 clinical trials) for the promotion ocular surface healing, increase corneal epithelial cell migration, and decrease corneal pro-inflammatory cytokine levels.

Finally, for the treatment of inflammation of bone tissue, **TPX-100,** a synthetic 23-mer peptide which structure is currently undisclosed, is under investigation (OrthoTrophix, 2021). This peptide is produced by OrthoTrophix and designed starting from Matrix Extracellular Phosphoglycoprotein (MEPE), a protein belonging to the family of Small Integrin-Binding Ligand N-linked Glycoproteins (SIBLING) (McGuire et al., 2018). Evidence shows that these proteins play key roles in the mineralization of bones and dentin without a clear knowledge of the mechanism of action. It has been shown that the bone shape of the knee undergoes unidirectional and irreversible change over time, with a much greater rate of change in osteoarthritic knees than in normal ones (OrthoTrophix, 2021). Such accelerated bone shape mutation starts much earlier than cartilage degeneration and appears to be the most reliable structural marker, to date, for onset of osteoarthritis (OA). Even if the mechanism of action is still undisclosed, TPX-100 has completed Phase 2 clinical trial for the treatment of the knee osteoarthritis, inducing the regeneration of normal tibiofemoral hyaline cartilage and highlighting a statistically significant correlation between stabilization of tibiofemoral cartilage and critical knee functions.

## Genetic and Hormonal Diseases

Genetic disorders can be caused by a mutation in one gene (monogenic disorder), by mutations in multiple genes (multifactorial inheritance disorder), by a combination of gene mutations and environmental factors, or by damage to chromosomes (National Human Genome Research Institute NHGRI, 2021). On the other hand, hormonal disorders may result from a problem in the glands themself, or because the hypothalamic-pituitary axis (interplay of hormonal signals between the hypothalamus and the pituitary gland) provides too much or too little stimulations (Emanuele and Emanuele, 1997). In this context, peptide-based treatments can be employed both as complementary supply of the lacking endogenous molecules (genetic disorders) or/and as interfering agents on the mechanisms responsible for their dysregulated production (hormonal disorders).

**HTL0030310** is a new synthetic peptide produced by Sosei Heptares which has recently completed Phase 1 of clinical trials for the evaluation of its safety and tolerability towards healthy subjects. Even though HTL0030310 structure is still undisclosed, Sosei Heptares designed this peptide as a selective SSTR5 (somatostatin 5) receptor agonist to treat endocrine disorders (Sosei Heptares, 2019) modulating the excess release of hormones from adenomas (benign tumors) of the pituitary gland. Highly elevated plasma levels of pituitary hormones result in a number of serious disorders including Cushing’s Disease, a debilitating endocrine disorder caused by the overproduction of the hormone cortisol.

On the other hand, **Vosoritide** is a synthetic 39-mer cyclic peptide produced by BioMarin Pharmaceutical and currently in Phase 3 of clinical trials for the treatment of achondroplasia, the most common form of disproportionate short stature in humans (Savarirayan et al., 2020). This condition is caused by an autosomal dominant mutation in the fibroblast growth factor receptor 3 (FGFR3), a gene that constitutively activates the mitogen-activated protein kinase (MAPK), which inhibits endochondral ossification (Savarirayan et al., 2019). Since C-type natriuretic peptide (CNP) and its receptor, natriuretic peptide receptor 2 (NPR2), are potent stimulators of endochondral ossification, it was observed that a continuous intravenous infusion of exogenous C-type natriuretic peptide restores the impaired bone growth. Vosoritide was designed as a cyclic analogue of C-type natriuretic peptide in order to increase the half-life in comparison with its endogenous form.

## Diabetes, Obesity, Short Bowel Syndrome and Hyperinsulinemia, the Proglucagon Legacy

Over the last decades, a series of synthetic peptides derived from the gut endocrine system were identified as promising treatments for metabolic diseases such as Type 2 Diabetes Mellitus (T2DM) and obesity. These pathologies still remain global health problems in continuous increase, known to reduce quality of life and to lead to serious complications (Bastin and Andreeli, 2019). The therapy of T2DM involves agents targeting body weight and simultaneously maintaining blood glucose control.

**FIGURE 5:**
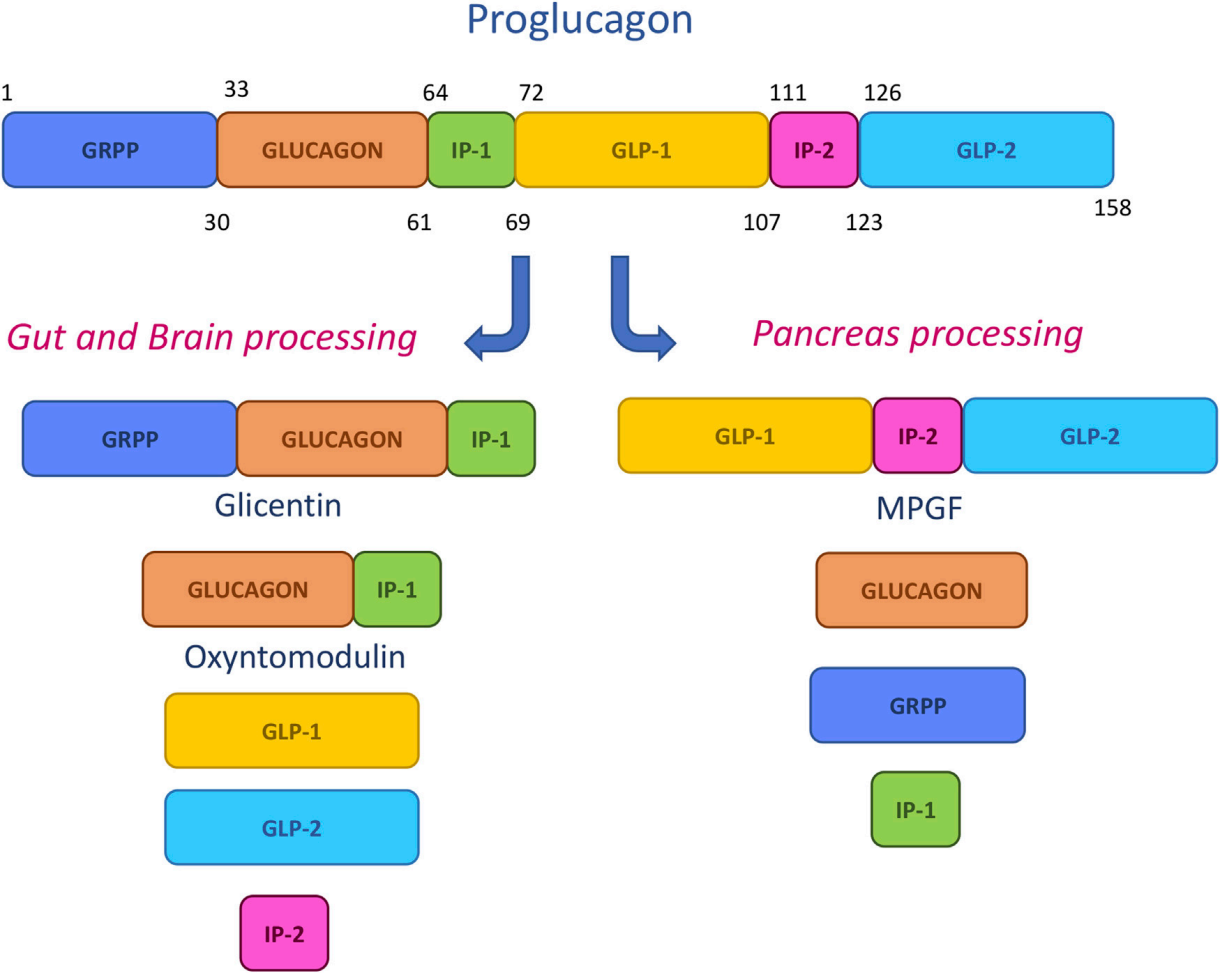
Tissue-specific proglucagon cleavage.

The main synthetic therapeutic peptides have been designed by mimicking the sequences deriving from the pro-hormone proglucagon metabolic cleavage. This 158-mer small protein is submitted in pancreas, gut and central nervous system to specific posttranslational processing by prohormone convertase enzymes, leading to the generation of shorter fragments (Figure 5). Among them, glicentin-related pancreatic peptide (GRPP), glucagon, intervening peptide-1 (IP-1) and intervening peptide-2(IP-2), major proglucagon fragment (MPGF), oxyntomodulin (OXM), glucagon-like peptide-1 (GLP-1) and glucagon-like peptide-2 (GLP-2) have a prominent role in glucose control, energy balance and gut homeostasis and were hence proposed as models for drug design (Lafferty et al., 2021).

### Glucagon Like Peptide-1 Receptor Targeting Peptides

One of the main biological targets for pharmaceutical action is the Glucagon like peptide-1 receptor (GLP-1R), a class BG-protein coupled receptor (GPCR), which is deeply involved in insulin secretion and homeostasis. Like other G-proteins, the GLP-1R is characterized by the seven transmembrane α-helices separated by three intracellular and three extracellular loops.

The GLP-1R activation is related to the secretion of glucagon like peptide 1 (GLP-1), an incretin glucoregulatory peptide released in the gut after food intakes. GLP-1 exists in two forms: GLP-1(7-37) and GLP-1(7-36) amide, with the latter being more abundant in circulation after eating. These peptides, interacting with GLP-1R, enhance the insulin secretion and normalize the glucose levels through the receptor signalling, *via* cyclic adenosine monophosphate (cAMP) production (Druker and Nauck, 2006). When the regulatory effect is complete, the GLP-1 is inactivated by proteolysis. Over this main effect, GLP-1 and its agonists can reduce the glucagon secretion, slow the gastric transit and decrease the energy consumption extending the effects on treatment of obesity. The key role of the GLP-1R/GLP-1 system is reflected by the efforts recently made in developing efficient GLP-1R peptide agonists for pharmacological treatment of T2DM (Sloop et al., 2018).

A great advance in the field was reached with the discovery of exendin-4 in the venom of the *Gila monster* lizard. Exendin-4 (or Exenatide, as it has been called its synthetic form) is a 39-mer peptide resistant to dipeptidyl peptidase 4 (DPP-4), which shows a better pharmacokinetic than GLP-1 thanks to a Gly^2^ residue in its sequence. Exenatide was approved by FDA in 2005 and is dosed as a twice daily injection (Donnelly, 2012). Currently the best in class drugs for T2DM and obesity are liraglutide and more recently the orally disposable semaglutide. These two molecules foster the research for better GLP-1 analogues with improved pharmacological profile.

Following these therapeutic needs, further modifications of the exenatide sequence led to slow-release compounds: an example is **PB-119**, a polyethylene glycosylated exenatide developed by PegBio and obtained by modification of a single amino acid in exenatide, replacing Ser^39^with Cys (Min Xu et al., 2019). Even if the mechanism of action is identical to that of Exenatide, the PEGylation on the analogue PB-119 reduces the excretion rate in the kidneys, and its increased molecular weight causes steric hindrance toward DPP-4, prolonging the half-life and allowing one weekly subcutaneous injection dose regimen (Cui et al., 2020). The same concept of improved pharmacokinetic and resistance to DPP-4 was declined also by Jiangsu Hansoh Pharmaceutical through the development of the GLP-1R agonist **Noiiglutide**, a 40-mer exenatide analogue, currently in Phase 2 trials as subcutaneous injectable agent. Structurally, the presence of an unnatural Aib (α-amino isobutyric acid) residue in position two should guarantee a better resistance toward enzymatic hydrolysis while the condensation with palmitic acid on Lys^1^-ξ-NH_2_ enhances the hydrophilicity (Wang et al., 2012).

The GLP-1R agonist roster includes also **G3215** (structure undisclosed), developed by Imperial College, that completed its Phase 1 trials in 2019 (Serrano et al., 2017).

As above described, GLP-1R agonist peptides are receiving an increasing interest, with several agents advancing the clinical phases. In comparison, less efforts have been focused on studying GLP-1R antagonists. Nevertheless, Eiger BioPharmaceuticals is developing **Avexitide** for treating post-bariatric hypoglycemia (PBH), a form of hyperinsulinemic hypoglycemia (HH), as a new liquid formulation of Exendin 9−39 intended for subcutaneous injection administration. PBH is a rare disease without approved pharmacotherapy, caused by abnormal growth in insulin secretion, which is attributed to exaggerated postprandial secretion and to the large increase in meal induced GLP-1 release (Craig, 2020). Therefore, GLP-1R antagonism could represent a potential therapeutic strategy for managing this condition. Avexitide is a 31-mer peptide that selectively targets and blocks GLP-1R, normalizing insulin secretion and thus decreasing postprandial hypoglycemia. Data released from a recent Phase 2 clinical trial showed that Avexitide produced consistent improvements in the incidence of hypoglycemia maintaining a good tolerability profile (Suzuki et al., 2020).

### Glucagon Like Peptide-1 Receptor/GPCR Glucagon Receptor Targeting Peptides

The clinical success of GLP-1R agonist peptides has prompted the search for agents that could combine the benefits of GLP-1 with those of other key gut hormones, in order to target multiple signalling pathways with a single molecular entity. Moreover, this emerging dual pharmacological approach may lead to increased metabolic action compared to monotherapies, and a number of these candidates are being evaluated in clinical trials (Bastin et al., 2019).

Glucose homeostasis is regulated by the combinatorial action of insulin and glucagon. While insulin and its therapeutic applications were widely studied, the glucagon hormone, secreted by the pancreatic α-cells, has been receiving attention only in the last decades. Its main action consists in a counter-regulatory effect toward insulin, inducing hepatic glycogenolysis and gluconeogenesis by targeting the class B GPCR glucagon receptor (GCGR) (Herr, 2012). The use of native glucagon in the therapeutic approach to hypoglycaemia is not easily practicable (Patil et al., 2020) for its very low stability in physiological formulations and its consequent high tendency to aggregate in few hours, after reconstitution (Hӧvelmann et al., 2018). Thus, the development of new analogues/agonists of the native glucagon peptide is necessary in treatment of hypoglycaemia. Zealand Pharma developed, **Dasiglucagon** (NDA) which will be launched on the market in the first trimester of 2021 as Zegalogue^®^, a ready-to-use formulation administered by single-injection pen applicator. To avoid aggregation problems and fibril formations and to render it physically and chemically stable in aqueous solution, Dasiglucagon was designed introducing seven substitutions in the 29 amino acid sequence of the native glucagon, with the insertion of an Aib residue (Pieber et al., 2020).

On the other hand, glucagon enhances energy consumption and acts as food intake regulator, making its analogues also interesting for glucose regulation in T2DM and obesity (Suzuki et al., 2020). GLP-1 and glucagon have a high sequence homology: this aspect encouraged the development of multiple molecules with an integrate pharmacological profile targeting both GLP-1R and GCGR in a dual-agonism mechanism, where glycaemia and weight control are accompanied by adipose tissue lipolysis and appetite suppression (Capozzi et al., 2018). The native gut hormone peptide Oxyntomodulin (OXM), secreted after food ingestion, exhibits an antidiabetic effect and drives weight loss acting on appetite and satiety by activating both GLP-1R and GCGR, even if showing lower activity compared to GLP-1 and glucagon alone. Thus, oxyntomodulin has been chosen as a model for the development of GLP-1R/GCGR dual agonists.

Within this class, **Pegapamodutide** (OPK880033), by OPKO Health, completed phase 2 for once-weekly administration in treatment of T2DM. This drug candidate consists in a PEGylated analogue of OXM and displays a long action since the presence of two PEG moieties on Cys^38^and Cys^39^ prevents enzymatic degradation (Sheetz et al., 2017) The resistance toward DPP-4 is also provided by the presence of two un-natural Aib residues along the sequence. In particular, Aib^2^ enhances potency and selectivity at the GLP1-R. (Connop et al., 2020).

In addition, MedImmune, a subsidiary of AstraZeneca, is developing **Cotadutide** (MEDI0382), presently in Phase 2 as once-daily, subcutaneous therapy, to treat overweight or obese patients with T2DM. This novel dual GLP-1R/GCGR agonist was designed to improve glycaemic control and to facilitate weight loss through combined effects on appetite and energy expenditure (Ambery et al., 2018). Structurally, MEDI0382 is a 30-mer synthetic linear peptide, designed as OXM analogue, wherein the sequence of oxyntomodulin has been C-terminally depleted of seven residues, while seven of the remaining amino acids have been changed. Its extended half-life was achieved by enhancing stability to peptidase degradation: Gln^20^ and Gln^24^ were replaced with residues not susceptible to deamidation, whilst Arg^17^ was substituted with Glu to decrease proteolysis. Moreover, the insertion of a palmitic acid side chain at Lys^10^ through a γ-glutamate moiety promoted its reversible binding to albumin (Henderson et al., 2016). In such a way, this degradation resistant peptide is able to exert an optimally balanced dual agonism, inducing in clinical trials’ patients an observed weight loss greater than that observed with GLP-1R alone.

Another long-acting GLP-1R/GCGR dual agonist, **BI456906**, is being developed by Zealand Pharma in collaboration with Boehringer Ingelheim. This peptide is an analogue of the natural OXM and is being investigated in Phase 2 for T2DM and obesity, (Zealand Pharma, 2020). Efficacy of BI456906 as a treatment for non-alcoholic steatohepatitis (NASH), the most severe form of non-alcoholic fatty liver disease (NAFLD), is also expected, due to the emerging successful approach for this pathology using dual GLP-1R/GCGR agonists.

In this field, **ALT-801** emerged as a novel peptide-based dual GLP-1R/GCGR agonist designed by AltImmune to treat the obesity and metabolic dysfunction caused by NASH. As observed in a preclinical model of the disease, ALT-801 induced significant weight loss compared to semaglutide, and accordingly the company recently announced the beginning of a Phase 1 clinical trial.

Its structure is based on a 29-mer sequence partially mimicking both GLP-1 for an improved weight loss and glucagon for restoring metabolic functions (Nestor et al., 2021). The insertion of an Aib residue was meant to prevent the proteolytic degradation, while a EuPort™ liposaccharide surfactant, formed by a D-glucoside linked to a methylene C18 chain, was introduced on Lys^17^ side chain to improve gastrointestinal tolerability. In addition, Glu^16^ and Lys^20^ side chains were conjugated to create a lactam ring, designed as a helix stabilizer to increase potency. These structural features afforded a high serum albumin binding, leading to a suitable once-weekly subcutaneous administration in patients (AltImmune, 2019). Finally, **LY3305677** (OXM3, IBI362) is an undisclosed peptide dual GLP-1R/GCGR agonist, reported by Ely Lilly as a third-generation analogue of Oxyntomodulin, that is currently under evaluation in Phase 2 clinical trials.

### Glucagon Like Peptide-1 Receptor/GIPR Targeting Peptides

Another strategy to treat diabetes and obesity with dual active peptides is exploiting the agonism toward incretin receptors GLP-1R and GIPR. GIPR is the natural target of the native gut hormone glucose-dependent insulinotropic polypeptide (GIP), secreted by the *K*-cells after food intakes. This incretin peptide can stimulate insulin and glucagon secretion, like GLP-1, but with a weaker efficacy on the regulation of the insulinotropic effect. Studies on the receptor-ligand interaction pointed out the key role of the native peptide N-terminus in GIPR activation. This moiety interacts with a binding pocket in the transmembrane receptor, while the central residues bind to the extracellular domain of the receptor. Accordingly, a tuning of the N-terminal moiety could modulate the agonism or the antagonism of the peptide (Gasbjerg et al., 2018).

Therefore, the GIP agonism has been identified as a pharmaceutical strategy for the treatment of T2DM, even if it seems to be less relevant than GLP-1 agonism. Synthetic dual incretin receptor agonists, nicknamed “twincretins,” have been investigated in the last years, with a few candidates actually in clinical development. The most advanced GLP-1R/GIPR agonist is **Tirzepatide** (LY3298176) from Eli Lilly, currently in Phase 3 trials. Tirzepatide is formulated as a synthetic 39-mer linear peptide, sharing 19 residues with native GIP. The peptide sequence incorporates the amidated C-terminus and two non-natural Aib residues at positions 2 and 13. Moreover, the structure is conjugated to a 20-carbon fatty diacid moiety *via* a hydrophilic linker connected to the Lys^20^ residue. This strategy was designed to promote albumin binding, prolonging Tirzepatide half-life to approximately 5 days and thus enabling once-weekly subcutaneous dosing regimen (Coskun et al., 2018). In phase 1 and 2 clinical trials, Tirzepatide produced significantly improved clinical efficacy, safety and tolerability in glucose control and weight loss compared with the GLP-1 agonist dulaglutide. GIPR binding affinity resulted comparable to that of native GIP, while a GLP-1R affinity five times lower than that of native GLP-1 was observed (Min and Bain, 2021).

It is worth mention that also the dual agonist peptide **SCO-094**, developed by Scohia Pharma in partnership with Takeda Pharmaceuticals (Clemmensen et al., 2019) belongs to this class of drug candidates. This molecule is being tested in both long-acting and oral formulations in Phase 1 with wide potential applications comprising diabetes, obesity and NASH.

Finally, the dual agonist **CT-868**, developed by Carmot Therapeutics, will enter in Phase 2 in the first half of 2021 as a promising treatment for diabetes, obesity and NASH (Kim and Kim, 2020). CT-868 dual agonist candidate was discovered using the Chemotype Evolution technology, as a peptide-small molecule hybrid compound, able to mimic the native GLP-1 hormone (Carmot Therapeutics Pipeline, 2021).

On the basis of the promising results of dual targeting peptides, some ternary agonists concurrently targeting GLP-1, GCGR and GIPR have been developed. The peptide **LY3437943** from Ely Lilly is the first member of this novel class of peptides, designed to be effective in T2DM and obesity treatment. The structure of this peptide is still undisclosed by the originator.

### Y Receptor Type 2 Targeting Peptides

Other potential therapies for obesity and associate pathologies involve PYY and amylin agonists.

Peptide YY (PYY) is a gut hormone that is co-secreted from the entero-endocrine L cells, together with GLP-1 and oxyntomodulin, in response to feeding (Khoo and Tan, 2020). Although it exists in two major forms, the full length PYY1-36 and PYY3-36, the latest is the most commonly biologically active circulating form. PYY3-36 is a 34-mer peptide released postprandially by the gut. Its secretion activates appetite suppression and food intake decrease by acting, in the arcuate nucleus of the hypothalamus, on neuropeptide Y receptor type 2 (Y2R), a member of the neuropeptide Y receptor family that belongs to the GPCR family (Srivastava and Apovian, 2018).

Early clinical studies showed that PYY3–36 infusion in obese patients induced suppression of food intake with good tolerability, and limited side effects. Anyway, since native PYY short half-life (8 min) affects clinical stability, various approaches have been planned to increase its resistance to proteolytic inactivation (Will et al., 2017). In this context, alternative delivery of PYY is a challenge. Recently, Gila Therapeutics has developed **GT-001**, a PYY3–36 analogue that started Phase 1 trials with orally available sublingual formulation (Acosta et al., 2019). Novo Nordisk’s pipeline also includes a novel analogue of the appetite-regulating hormone PYY, namely **PYY-1875**, whose structure is still undisclosed. This peptide is currently in Phase 1 and is intended as subcutaneous injection for once-weekly treatment (Novo Nordisk, 2020).

Amylin is a 37-mer peptide hormone mainly produced in the pancreatic beta cells and co-secreted with insulin during a meal. Amylin has a well-established role as a satiety signal; it acts by reducing food intake and postprandial glucagon secretion *via* binding to human amylin receptors (AMY) in specific areas of the brain (Zakariassen et al., 2020). AMY subtypes are GPCR consisting of a calcitonin receptor (CTR) and one of three receptor activity-modifying proteins (RAMP). Because of their mechanism of action, amylin mimetics are novel targets of study as anti-obesity drugs and several approaches (PEGylation, glycosylation or albumin binding) have been explored to extend their half-life and reduce their administration frequency. Novo Nordisk is currently testing **AM833** in phase 2 trial, a long-acting acylated analogue of the human amylin hormone, in once-weekly subcutaneous administration, AM833 has been evaluated in combination with the GLP-1 analogue semaglutide in a phase 1 clinical trial (Mathiesen et al., 2020).

### Glucagon-Like Peptide-2 Receptor Targeting Peptides

Glucagon-like peptide-2 receptor (GLP-2R) is a GPCR superfamily member expressed in the gastrointestinal tract and, belonging to the family of seven trans-membrane receptors, it is closely related to GCGR and GLP-1R. Through binding to glucagon-like peptide-2 (GLP-2), GLP-2R directly inhibits apoptosis and increases intestinal growth by stimulating cell proliferation in response to ligand activation. GLP-2 is a 33-mer hormone released by the enteroendocrine intestinal L-cells in response to nutrient ingestion (Brubaker, 2018).

Although the mechanisms by which GLP-2 mediates its effects still remain not completely understood, it is an important regulator for stimulating intestinal growth, increasing absorption, promoting healing and maintaining epithelial integrity, in both normal humans and patients with intestinal failure consequent to massive intestinal resection (short bowel syndrome, SBS). Indeed, patients with SBS might have impaired postprandial GLP-2 secretion, which is instead required for optimal intestinal adaptation. The use of GLP-2 as a therapeutic agent is limited because of its very short circulating half-life (approximately 7 min) due to cleavage by DPP-4 (Hargrove et al., 2020). Consequently, pharmacologically active GLP-2 analogues with a longer half-life and reduced clearance are under development against SBS, including phase 3 agents **Apraglutide** (VectivBio) and **Glepaglutide** (Zealand Pharma).

Glepaglutide is a highly potent GLP-2R selective 39-mer peptide, with a long-acting effect and an effective plasma half-life of approximately 50 h, allowing a less-than-once daily administration. Furthermore, it offers a more convenient dosing form *via* a ready-to-use autoinjector device, which removes the requirement for reconstitution from lyophilized powder and allows the formation of a subcutaneous depot at the site of the injection, from which it could be slowly released into the circulation (Naimi et al., 2019). Glepaglutide differs from native GLP-2 through the insertion of a C-terminal tail consisting of six lysine residues that modify the charge state aiding solubility and physicochemical properties. The N-terminal region was optimized by changing five amino acids for improving pharmacokinetic and potency properties, and by replacing other four residues with alanine in an internal region, ending in a globally nine amino acid substitution.

Apraglutide is another highly selective, potent GLP-2R agonist peptide, composed by a 33 amino acid sequence, with a molecular structure designed to preserve optimal pharmacological activity while increasing the half-life compared to native GLP-2 or other GLP-2 analogues (Martchenko et al., 2020). Specifically, native GLP-2 was modified at positions 2, 10, 11 and 16 with Gly, Nle, D-Phe and Leu respectively, and with a C-terminal amidation. The few amino acid substitutions translate into a superior pharmacokinetic profile that results in an exceptionally low clearance and in a high plasma protein binding, thus enabling a long *in vivo* elimination half-life after subcutaneous administration (30 h), without necessity of conjugation or other peptide modifications. Moreover, Apraglutide displays a good stability against DPP-4, allowing the possibility for only once-to-twice weekly treatment (Slim et al., 2019).

The effect of the native GLP peptides was investigated in SBS patients either as a selective agonist (GLP-1R or GLP-2R) or as a dual GLP agonist combination therapy, with the latter imparting superior efficacy and better patient outcomes compared to each single agent alone (Suzuki et al., 2020). This rationale supported the use of dual GLP receptor co-agonism for the treatment of metabolic and gastrointestinal diseases, even if clinical studies are still awaiting to confirm the therapeutic efficacy of its combination. Following this approach, Zealand Pharma is currently developing **Dapiglutide**, previously referred to as ZP7570. Even if its structure is still undisclosed, the company announced that this molecule completed Phase 1 trials in 2020 and showed a good safety and tolerability profile in healthy volunteers together with a plasma half-life allowing for once-weekly dosing (Zealand Pharma, 2020).

## Cardiovascular System and Hypertension

Cardiovascular diseases (CVD) are among the leading causes of death worldwide, taking an estimated 17.9 million lives each year. According to the World Health Organization (WHO), four out of five CVD deaths are due to heart attacks and strokes, and one third of these deaths occurs prematurely in people under 7 years of age (WHO, 2021). In the last years, proteins and peptides with unique biological activity and metabolism have successfully caught the attention of researchers as an alternative treatment for cardiovascular and hypertension diseases and several are still under clinical study waiting to be approved.

**Ularitide**, an atrial peptide agonist, was developed by Cardiorentis, a private biopharmaceutical company, specialized in the treatment of cardiovascular diseases, as an intravenous treatment for acute heart and kidney failure, and has completed the Phase 3 in 2018 (Emani et al., 2015). Ularitide is the chemically synthesized form of urodilatin, a human endogenous natriuretic peptide produced in the kidneys with the aim to regulate fluid balance and sodium homeostasis. This 32-mer-containing peptide has the same sequence of the 28 amino acid-containing atrial natriuretic peptide (ANP), except for the addition of four amino acids at the N-terminal extension. The crystal structure of ANP bound to the receptor showed that the complex contains two NPR-A molecules bound with one molecule of ANP and, since the ANP molecule has no internal symmetry, binding to the receptor is asymmetric and occurs through two different binding sites present in each NPR-A monomer (Ogawa et al., 2004). Ularitide binds primarily to the extracellular domain of natriuretic peptide receptor-A (NPR-A) which is expressed in the heart, kidney and other organs and activates the intracellular guanylate cyclase domain of the receptor (Anker et al., 2015). Guanylate cyclases catalyze the conversion of guanosine 5′ -triphosphate to cyclic guanosine-3′, 5′ -monophosphate (cGMP) causing vasodilation through vasorelaxation of smooth muscle cells and natriuresis and diuresis through inhibition of sodium reabsorption.In the treatment of stroke-associated pathologies, cationic arginine-rich peptides (CARPs) represent a new emerging class of neuroprotective agents with multimodal cytoprotective actions. Among them, **NA-1**, originally named TAT-NR2B9, is a lead compound being developed by NoNO for the treatment of stroke, traumatic brain injuries and subarachnoid hemorrhage and is currently in Phase 3 clinical trial. NA-1 is a 20-mer peptide consisting in a sequence (KLSSIESDV) derived from the intracellular terminal carboxylic region of the N-methyl-D-aspartate (NMDA) receptor NR2B subunit protein, fused to the cationic arginine-rich cell penetrating peptide TAT (YGRKKRRQRRR), which facilitates the passage across the blood brain barrier (Ballarin and Tymianski 2018; Meloni et al., 2020). The NR2B9 9-mer peptide was selected to inhibit the postsynaptic density protein-95 (PSD95) adaptor protein, which binds to the NR2B subunit, and thereby blocks downstream cell signalling associated with overstimulation of the NMDA receptor.

**CN-105**, a small 5-mer apolipoprotein E (apoE) mimetic peptide, rich of arginine residues and deriving from the receptor binding region of apoE, is currently under development by AegisCN (formerly CereNova). This peptide is in Phase 2 for the treatment of hemorrhagic stroke and intracerebral hemorrhage. CN-105 has been developed based on the amino acids present in the parent neuroprotective peptide COG133, comprising the heparin binding and LDL receptor binding domains within the apoE (Meloni et al., 2020). ApoE is a multifunctional 299-mer protein that reduces neuroinflammation and mediates adaptive responses following ischemic and traumatic brain injury. However, the intact apoE holoprotein does not cross the blood-brain-barrier (BBB) limiting its therapeutic potential. CN-105 has the advantage of an increased CNS penetration and has demonstrated efficacy in experimental intracerebral hemorrhage, leading to an improvement of functional and histological outcomes after experimental ischemic stroke, and reducing microglial activation (Tu et al., 2017; Yeh et al., 2020).

The last peptide still in clinical trials for the treatment of cardiovascular disease through protein-protein interactions is **Pemziviptadil** (PB1046). PB1046 is a novel, subcutaneously injected vasoactive intestinal peptide (VIP) analogue, developed by PhaseBio Pharmaceutical that is currently tested in Phase 2 for the treatment of pulmonary arterial hypertension (PAH). VIP is a 28-mer peptide hormone that activates VPAC1 and VPAC2 receptors in the pulmonary vasculature and has been shown to relax pulmonary vascular smooth muscle, to neutralize pulmonary vasoconstrictors, and to inhibit cell proliferation (Chappe et al., 2014). The short half-life of VIP renders this peptide impractical as a pharmaceutical agent and modified versions of VIP are therefore needed to render the agent therapeutically useful (Sadeghi et al., 2015). PB1046 is 29-mer peptide linked to elastin-like polypeptide (ELP) biopolymer. The ELP component comprises structural peptide fragments that are related to the elastin protein. Such modified sequences are useful for improving important properties as absorption profile and circulating half-life (PhaseBio, 2020).

## Neurodegenerative Diseases

Neurodegenerative diseases are the result of a continuous process based on degenerative cell changes, which increasingly deteriorate over time. Aggregation of pathogenic proteins, mitochondrial dysfunction, oxidative stress, transcriptional dysfunction and apoptosis play an important role in the development of neurodegenerative disorders such as Parkinson’s disease (PD), Alzheimer’s disease (AD) and Amyotrophic lateral sclerosis (ALS) (Emerit et al., 2004). Up to now, no novel disease-modifying therapies have been shown to provide significant benefits for patients who suffer from these devastating disorders. Therefore, early diagnosis and the discovery of new drugs able to bind selectively specific targets are increasingly required on the market.

**Davunetide** (NAP, AL-108), is an intranasally administered, 8-mer peptide fragment deriving from the activity-dependent neuroprotective protein (ADNP), actually in Phase 1 for the treatment of progressive supranuclear palsy (PSP). Davunetide has preclinical evidence for neuroprotective, neurotrophic and cognitive protective properties by promoting microtubule stabilization. (Javitt et al., 2012). The protein tubulin, constituting the microtubule backbone, was shown to be the target of NAP, recently reported as an enhancer of the interaction between microtubules and microtubule associated proteins, and microtubule polymerization under conditions of zinc intoxication (Magen and Gozes, 2014). NAP has also been reported to reduce levels of neurotoxic and pro-inflammatory factors, such as nitric oxide (NO) and tumor necrosis factor (TNF) by blocking microglial activation. The microtubules end-binding (EB) proteins have been identified as the targets of NAP, through interaction of its SIP domain. The binding of davunetide to its receptor has been recently predicted using structural alignment of EB3 to EB1-EB3 complex with another peptide ligand (MACF) (Vaisburd et al., 2015)

**NLY01** (Neuraly Inc.) is a pegylated long-acting analogue of exendin-4, (Yun et al., 2018). In the Phase 1 study, NLY01 was well-tolerated, and showed a half-life three times higher than shorter-acting GLP-1R agonists, which are often limited by side effects. In 2020 the approval of an investigational new drug application (IND) to initiate a Phase 2b clinical trial of NLY01 in patients with AD and a Phase 2 trial in patients with PD were announced.

In a previous study, it was shown that NLY01, through a favorable blood brain barrier penetration, binds upregulated GLP-1R, blocking pathological activation of microglia in animal models of neurodegenerative diseases, including Parkinson’s (Sterling et al., 2020).

Myasthenia gravis (MG) is another serious degenerative disease, a chronic auto-immune condition in which auto-antibodies attack specific proteins in the neuro-muscular junction, resulting in muscle weakness and fatigue. **Zilucoplan** is a small synthetic 15-mer macrocyclic peptide by UCB Pharma, currently in Phase 3 clinical trial for MG, acting as a potent inhibitor of the terminal complement protein C5, with potential anti-inflammatory and cell protective activities (UCB Pharma, 2020). Upon subcutaneous administration, Zilucoplan binds to complement protein C5, blocking C5 cleavage into C5a and C5b and preventing the C5b-dependent assembly of the membrane-attack complex (MAC). Zilucoplan also inhibits the interaction between C5b and C6, thereby further blocking MAC assembly. (Gorman et al., 2021; Duda et al.,2020).

Last but not least, **GM604** (GM6, Alirinetide) is a cationic linear peptide drug that has been developed by Genervon Biopharmaceuticals and is in Phase 2 clinical trial for ALS disease. The peptide consists of 6 amino acids, representing a subunit of the endogenous 33-mer peptide known as “motoneuronotrophic factor” (MNTF1) (Swindell et al., 2018). Preclinical tests suggest that MNTF regulates CNS biological functions, including neuronal differentiation, axonal regeneration, reinnervation, and inflammation and apoptosis, providing both neuroprotective and neuro-regenerative therapeutic effects. GM604 is expected to have a complex mechanism of action potentially involving stimulation of multiple receptors, signaling cascades and downstream gene expression responses (Ko et al., 2017). GM604 does not selectively interact with a single target, but rather interacts with multiple receptors linked to diverse pathways. These include insulin receptor, Notch receptors one to four and smoothened frizzled class receptor. Stimulation and activation of these and other receptors by GM6 is associated with the expression of thousands of genes leading to microtubule stability, synaptic transmission and axon guidance (Genervon, 2021).

## Bacterial and Viral Infections

Infectious diseases, caused by many pathogens, including bacteria, viruses, fungi, and parasites are extremely common worldwide, although a huge number of antibiotics and antiviral drugs are available on the market. Mutation of infectious microorganisms and development of resistant strains has to be continuously faced with new medicines and for this reason innovation if this area is a need. Up today, three peptides are in clinical phase with the purpose to treat these pathologies. Due to SARS-CoV-2 virus pandemic diffusion, in the last year all anti-infective peptides entered clinical trial to verify their possible use for this infection. Anyway, since these repositioning studies are not based on a medicinal chemistry approach targeting protein receptors, reliable results are still not available. For these reasons, we decided to exclude them from this overview.

**Nangibotide** (LR12) is a chemically synthesized 12-mer peptide derived from residues 94 to 105 of TREM-like transcript-1 (TLT-1). LR12, launched by Inotrem, is a specific TREM-1 inhibitor, interfering in the binding of TREM-1 and its ligand (Cuvier et al., 2018; Francois et al., 2020). TREM-1 is an amplifier of the innate immune response by synergizing with toll-like receptors and is a crucial mediator of septic shock. LR12 blocks TREM-1 by binding to its ligand and provides protective effects during sepsis such as inhibiting hyper-responsiveness, organ damage and death, without causing deleterious consequences (Inotrem, 2020). The protective effects of modulating TREM-1 signaling are also evident in other models of inflammation such as pancreatitis, hemorrhagic shock and inflammatory diseases. The drug is currently in a Phase 2b trial for treating septic shock and results are expected by the second half of 2021.

**Reltecimod** (AB103, p2TA; CD288–15) was discovered by Atox Bio as a drug candidate for the resolution of organ dysfunction or failure by attenuating the dysregulated immune response frequently seen in patients with necrotizing soft tissue infection (NSTI). It is an synthetic analogue peptide with homology to the fragment 8–15 of the T-lymphocyte receptor CD28. With its novel mechanism of action, it binds to the dimer interface of the costimulatory receptors and interferes with B7-2/CD28 engagement expressed on T-cells, thereby modulating the acute inflammation that leads to systemic organ failure (Kaempfer, 2018). Reltecimod completed the clinical trial process and its approval by regulatory organs is currently ongoing, suggesting the launch on the market in the next future. (Pipeline Overview AtoxBio, 2021).

**FIGURE 6:**
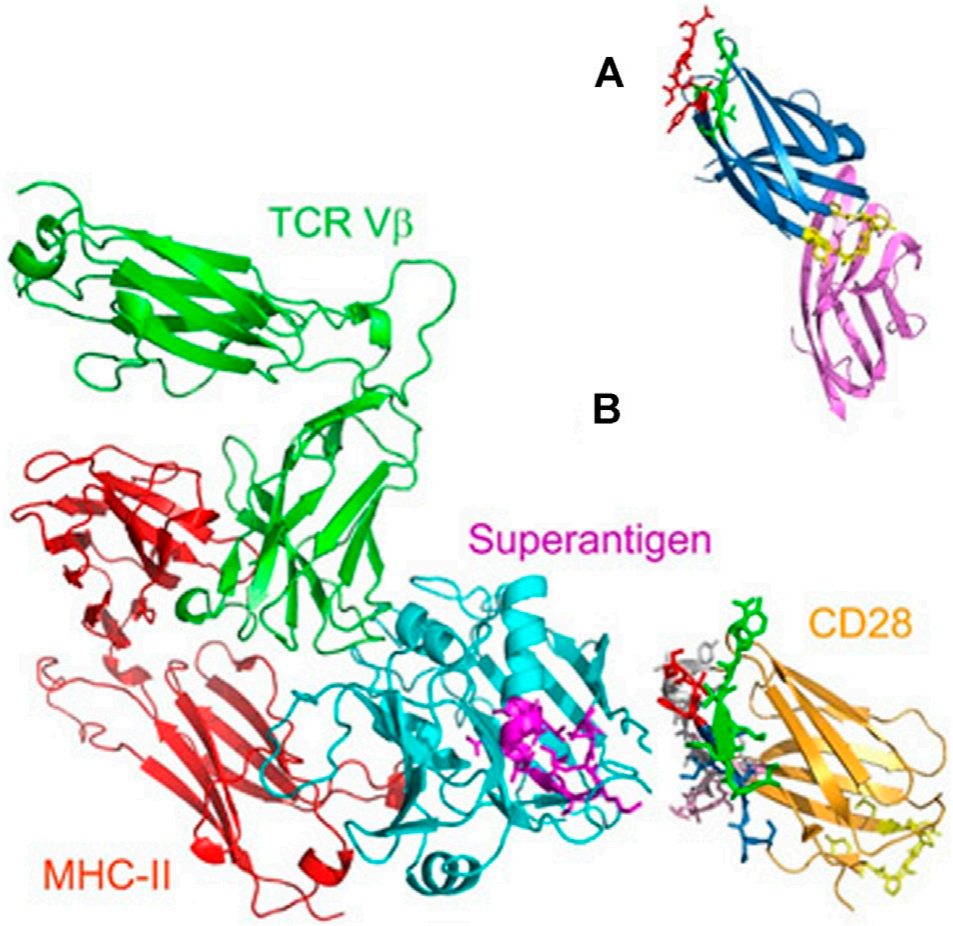
**(A)** Predicted CD28 dimer interface domain **(B)** Model for superantigen action through binding to CD28 (adapted from Arad et al., 2011).

To understand the binding of bacterial superantigen toxins to the CD28 homodimer interface, reltecimod (SPMLVAYD) was explored, being a fragment of the CD28 dimer interface domain predicted from alignment with CTLA-4 (Figure 6A). In the available structure for CD28, reltecimod overlaps with the dimer interface, taking part to the complex functional superantigen binding site in CD28. The peptide competes with cell-surface CD28 for the β-strand/hinge/α-helix domain in the superantigen, in a site that is remote from other proteins (MHC-II, TCR Vβ) binding sites (Figure 6B).

Blocking the access of a superantigen to CD28 is sufficient to block acute inflammation lethal toxic shock (Arad et al., 2011)

**C16G2** was developed by Armata Pharmaceuticals thanks to the design of a novel platform for the treatment of bacterial infections called “Specifically Targeted Antimicrobial Peptide (STAMP) platform”. STAMPs are designed by mining bacterial genomes for targeting domains that confer pathogen-specific killing activity. Therefore, C16G2, targeting *S. mutans* in the dental caries, was created by matching a targeting domain for effective accumulation on the *S. mutans* cell surface, as the competence-stimulating peptide C16 pheromone, with a killing domain, that is the G2 sequence as a truncated version of the broad-spectrum killing peptide novispirin G. A flexible tri-glycine sequence links the two regions. C16G2 has finished the Phase 2 clinical trials demonstrating an acceptable safety and tolerability profile of the drug and a selective reduction of S*. mutans* in the oral cavity. However, the company elected not to proceed to Phase 3 to seek alternatives to lower STAMP cost of goods (Kaplan et al., 2011).

## Miscellaneous

In this section several peptides targeting different diseases have been collected, since they cannot be classified in one of the specific pathological areas described in this review.

**LJPC-401** is a synthetic human hepcidin peptide, developed by La Jolla Pharmaceutical Company for the potential treatment of conditions characterized by iron overload. In healthy individuals, hepcidin prevents excessive iron accumulation in vital organs, such as the liver and heart, where it can cause significant damage and even result in death.

The feedback circuitry between hepcidin and iron levels in the body ensures systemic iron homeostasis. Hepcidin reduces the concentration of ferroportin-1 receptor (FPN-1) on the cell surface thereby inhibiting the entry of iron into plasma. Hepcidin binds FPN in a central cavity between the N and C domains, acting as a molecular cap to hinder the iron efflux pathway. The network of polar and hydrophobic interactions of the peptide with FPN has been assessed by evaluating decrease in binding affinity due to specific mutations (Billesbølle et al., 2020).

Binding of hepcidin to FPN-1 induces a modification in the receptor, leading to its internalization and degradation in lysosomes. In this way, the export of iron from reticuloendothelial macrophages, hepatocytes, and duodenal enterocytes is blocked (Casu et al., 2018). Hepcidin deficiency is a common feature of hereditary hemochromatosis (HH) and LJPC-401 has completed Phase 2 clinical trials for this therapeutic target, representing a “replacement therapy” to supplement inadequate hepcidin levels (Chawla et al., 2019).

**OXE-103** (Oxeia Biopharmaceuticals) is a synthetic human ghrelin, an endogenous hormone, to treat concussions/mTBI. OXE-103 freely crosses the blood-brain-barrier and helps stabilizing metabolic and energy brain dysfunction following a concussion. OXE-103 uniquely targets the hippocampus region of the brain, an important area for cognition and memory. Treatment with OXE-103 has been shown to restore normal energy metabolism, and to reduce the toxic effects of reactive oxygen species that form in low energy states. In neurons, the uncoupling protein-2 (UCP2) stabilizes mitochondria by responding to sublethal stress. The exact neuroprotective role of ghrelin is still unclear but UCP2-dependent mitochondrial stabilization effects in thalamic neurons have been suggested (Lopez et al., 2014). A Phase 2 study is running with the goal to reduce symptom burden with OXE103 treatment (Oxeia Biopharmaceutical, 2021).

**Solnatide**, is a synthetic cyclic 17-mer peptide whose molecular structure mimics the lectin-like domain (TIP) of human tumor necrosis factor (TNF) (Willam et al., 2017a, b), associated with high altitude pulmonary edema (HAPE) and adult respiratory distress syndrome (ARDS). Solnatide, also known as AP-301, has been developed by Apeptico Forschung und Entwicklung and is about to enter in Phase 2b in the treatment of various pulmonary diseases. Solnatide activates the lung epithelial sodium channel (ENaC), by directly binding to the crucial alpha-subunit of the channel and by stabilizing its open state, thus enhancing sodium ion uptake. The oligosaccharide-binding property of the TIP domain of TNF plays an important role in the mechanism by which TNF and solnatide interact with and activate ENaC, although the exact nature of this interaction is not yet understood.

**TAK639** (Takeda) is a synthetic 9-mer linear peptide which has completed phase 1 clinical trials for the treatment of Primary Open-angle Glaucoma (POAG) and intraocular pressure (IOP) (Savinainen et al., 2019). It is estimated that in 2020 more than 75 million people worldwide have suffered from ocular glaucoma; in particular, POAG is a complex optic neuropathy characterized by atrophy of the optic nerve and retinal ganglion cells and their axons, leading to irreversible blindness. Clinical evidences indicate the key role of expression and function of the natriuretic peptide network in several ocular systems, including in human trabecular meshwork. C-type natriuretic peptide (CNP) is the most potent in the class at lowering IOP in rabbits and further studies have demonstrated the presence of functional natriuretic peptide receptor-B (NPR-B) in the eyes of these animals. However, this promising treatment is affected by administration issues owing to the difficulties of atrial natriuretic peptides in penetrating the cornea to lower IOP. To overcome this drawback, **TAK639** was designed as a cornea-permeable CNP derivative, exhibiting increased production and cellular concentrations of cyclic guanosine monophosphate (cGMP), able to lower IOP (Martin V. et al., 2020).

## Current Challenges and Future Perspectives in Peptides Development as Therapeutic Agents

The details of the industrial drug discovery and development process are normally performed under a very high degree of confidentiality. Most of the time, the structure is kept secret as long as possible and Markush structures are used in product patents, not only to enlarge the scope of the claim, but also to cover the real drug candidate formula. The structure of 11 of the 58 peptides that we have tracked down was not disclosed. In addition, for what concerns heterologous peptides most of the time also the binding mode, the hot spots of the protein-protein interaction, the modelling and the SARS are kept confidential to delay as much as possible potential competitors, since generation of “me-too” structures of peptides is quite simple.

Heterologous peptides for PPI are normally identified *via* a normal small molecule drug discovery approach, with the target to identify hot spots mainly via peptides library screening. Once the hit is identified, the structural changes have the target to shorten as much as possible the peptide length, to increase and stabilize the biological activity, to crystallize receptors/ligands or their fragments and to clearly identify the hot spots generating a docking model. The lead optimization to get the drug candidate will further optimize activity and selectivity, and will screen the amino acids that can be used to improve the pharmacological profile.

The approach to native/analogous peptides is completely different since, in this case, the active peptide sequence is normally well known as well as the protein-protein interaction. The critical issue is establishing the relationship of a specific PPI with a disease. Bioinformatics and a better understanding of human molecular physiology have a key role in decreasing time and cost in respect to experimental approaches for the identification of new targets (Muttenthaler et al., 2021). Starting from the native sequence, that has been already identified by nature, the challenge of the medicinal chemist is then similar to the one of heterologous peptides, with the target to file a product patent that identifies a series of new structure in a very competitive environment.

**FIGURE 7:**
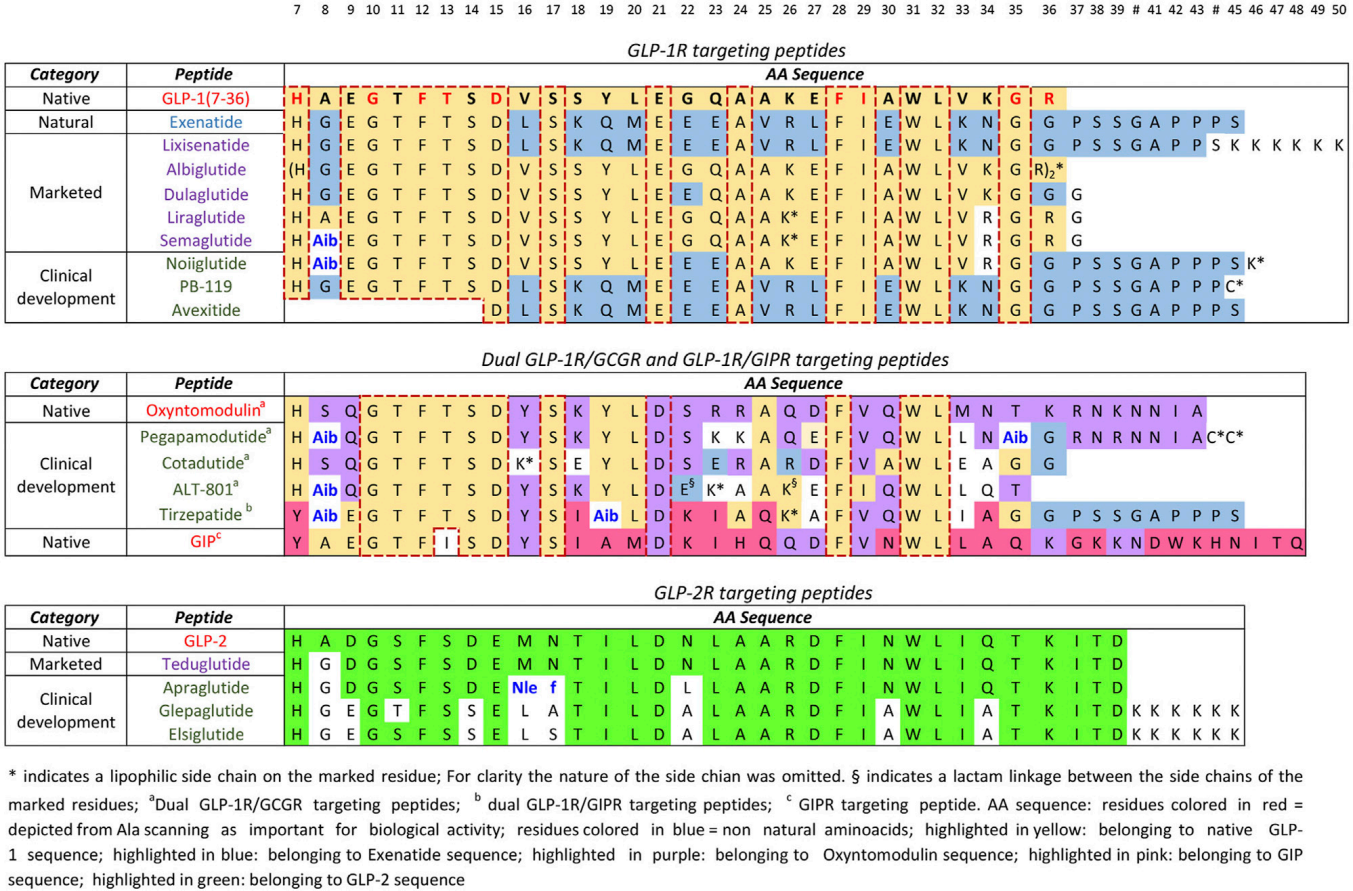
Alignment of analogue peptides with the corresponding native ones in T2DM/obesity and SBS treatment.

The most remarkable example of native/analogues peptides development is that of GLP-1R agonists for type-2 diabetes and obesity. The molecules in clinical trials have been already discussed in paragraph five, however, this segment, that is by far the most important PPI in terms of potential market and industrial competition, can be used for a critical evaluation. The first in class GLP-1R agonist for type-2-diabetes mellitus, exenatide, was approved by the FDA in 2012. This molecule is the synthetic version of exendin-4, a polypeptide found in the salivary excretion of the Gila Monster, that has a 53%

homology with GLP-1. While GLP-1 half-life is 1.5–5 min, exenatide is around 2.4 h. Since then, additional five molecules with a very high homology for GLP-1 or exenatide (>90%) have been approved and among them there are also the blockbusters liraglutide and semaglutide. From the alignments reported in Figure 7, it is easy to see that the amino acids that are critical for GLP-1R binding (Dods and Donnelly 2016), identified by site direct mutagenesis and Ala scanning (Knudsen and Lau 2019) have been conserved in all peptides (residues 7, 10, 12, 13, 15, 28, 29, 35, 36).

In fact, all modifications introduced in GLP-1 sequence and in the structures of dual inhibitors of GLP-1R/GIPR and GLP-1R/GCGR have the target to increase the pharmacological profile without perturbing the binding efficiency. With the only exception of liraglutide, in all the molecules already approved and in clinical trials, the Ala8 of GLP-1, that is sensitive to dipeptidyl peptidase-4 (DPP-4), has been replaced by Gly, Aib or Ser. In addition, side chains, known to prolong half-life and potentially increase oral bioavailability, were introduced in six of the seven molecules in clinical trials and have been attached to amino acids that are not critical for GLP-1R binding (residues 16, 23, 26, 36, 44, 45, and 46). From the available data semaglutide has still the record in terms of half-life (7 days). The overall development of these drugs has been largely described and the new molecules in clinical trials have followed a similar development path. From a scientific point of view, it will be interesting to monitor the evolution of dual and triple inhibitors in clinical trials to understand if these approaches will deliver any real therapeutic advantage respect to the current blockbusters.

Still serendipity can play a major role, and a typical example is Drucker’s discovery of the function of GLP-2 in the intestine that ultimately resulted in the commercialization of Teduglutide for the treatment of short bowel syndrome (Drucker 2019). The natural GLP-2 has a very short half-life, 7 min, while in Teduglutide the substitution of alanine with glycine at position two results in a peptide resistant to degradation by DPP-4 achieving a half-life of 2 h necessary for a daily treatment, in analogy with the GLP-1 analogues series (Marier et al., 2010). In other words, when the biological function of PPI is understood, the drug development is straightforward.

Therapeutic proteins (TP) have a consistently higher success rate than small molecules (Smietana et al., 2016). Peptides have performances similar to TP mainly because of the contribution of native/analogues and their high level of selectivity, that avoids out of target toxicity. On the other hand, heterologous peptides success rate is more similar to the one of small molecules and the target is, again, a very high level of selectivity, limiting the toxicological assessment to immunogenicity. When a market is saturated the impact of management and market considerations start to have a great impact on the attrition rate. Diabetes and obesity attracted the attention of several companies, since this is by far the most important market segment from a turnover point of view. We have described several native and analogue peptides that are targeting GLP-1R, GLP-1R/GCGR, GLP-1R/GIPR, Y2R actually in the clinic. However, there are already several peptides approved in this segment and, by the time the new ones will be approved, several potent and effective drugs will become generics. Therefore, the success rate of the molecules in the clinic will be not only related to efficacy but also by market positioning.

## Conclusion

The PPI modulation is a fertile area for peptide development as shown by the overall peptide market and by the large pipeline discussed in this paper. The herein reported overview shows that targeting PPI represents a successful approach for a number of different pathologies, since the network of human interactome regulates all the physiological cascade events and the related disfunctions. The availability of powerful synthetic methodologies is able to sustain medicinal chemists in the exploration of the molecular space for the design of new structures with an improved pharmacological profile. From a technological standpoint, innovation is now needed in the greening of synthetic technologies to accomplish sustainability.

Although the number of peptides entering clinical trials pathways is always increasing, a huge number of PPI are still unexplored or undisclosed. In fact, it is important to underline that, with the exception of cancer related pathologies as well as diabetes/obesity, the number of molecules in Phase 1 is very limited. The drug discovery and development process and the regulatory and market dynamics of therapeutic peptides will evolve in the near future also because of the potentially enormous impact of PPI. In conclusion, behind the PPI understanding there is the Holy Grail of protein-protein cross talk alphabet that will open up a new era in the pharmaceutical development of safe and effective drugs where the peptide modality will play a major role.
