# Supplementary material for: Therapeutic Peptides Targeting PPI in Clinical Development: Overview, Mechanism of Action and Perspectives
Source: Front Mol Biosci. 2021 Jun 14;8:697586. doi: 10.3389/fmolb.2021.697586 (PMC8236712; doi:10.3389/fmolb.2021.697586)
Supplement: Supplementary file 1 [file DataSheet1.PDF]

Therapeutic peptides targeting PPI in clinical development: overview, mechanism of action and perspectives

Walter Cabri, Paolo Cantelmi, Dario Corbisiero, Tommaso Fantoni, Lucia Ferrazzano, Giulia Martelli, Alexia Mattellone and Alessandra Tolomelli*

Department of Chemistry "Giacomo Ciamician", Alma Mater Studiorum University of Bologna, Via Selmi 2, 40126, Bologna (ITALY

*** Correspondence:**
Corresponding Author
alessandra.tolomelli@unibo.it

Keywords: oligopeptides, clinical trials, protein-protein interactions, mode of action, route of administration

SUPPORTING MATERIAL

Table 1S: Peptides with protein-protein interaction against specific tumors under clinical trial.

Name	Company	Biological Target	Target Disease	Clinical Trial Identifier	Clinical Phase	Structure[b]	AA(UAA)	CLASS[b]	RoA
**Dolcanatide** (SP-333)	Bausch Health Companies Inc; National Cancer Institute	Guanylyl Cyclase-C Receptors	Preventing colorectal cancer	NCT03300570<br>NCT01983306	1<br>2	nDEC(1)ELC(2)VNVAC(1)TGC(2)l	16	A	Oral
**BBT-401-1S** (BBT-401)	Bridge Biotherapeutics Inc.	Pellino-1-Protein	Ulcerative Colitis	NCT04596293	2	U	4	H	Oral
**Elsiglutide** (ZP-1846)	Zealand Pharma	GLP-2 Receptor	Adjuvant therapy for Induced Diarrhea in colorectal cancer patients	NCT02383810	2	HGEGSFSSELSTILDALAARDFIAWLIATKITDKKKKKK	39	A	SC
**Ozarelix** (D-63153)	Spectrum Pharmaceuticals	Gonadotropin-Releasing Hormone Receptor	Prostate Cancer	NCT01252693	2	Ac-D-2Nal-D-Phe(4-Cl)-D-3Pal-S-N(Me)Tyr-D-hCit-Nle-RPa-NH_2	10(6)	A	SC
**Fexapotide** (NX-1207)	Nymox Pharmaceutical Corporation	-	Benign Prostatic Hyperplasia/Lower Urinary Tract	NCT01620515<br>NCT01846793	2<br>3	IDQQVLSRIKLEIKRCL	17	U	I-PROSTAT
**Foxy-5** (Foxy 5)	WntResearch AB	Receptor of Wnt5a	Metastatic Prostate, Breast & Colon Cancer	NCT03883802<br>NCT02655952	2<br>1	N-Formyl-MDGCEL	6	A	IV
**VT1021**	Vigeo Therapeutics	CD36 And CD47	Solid Tumor	NCT03364400	1	U	5	U	U
**EP-100**	Esperance pharmaceuticals Inc.	Luteinizing Hormone Releasing Hormone Receptor	Ovarian Cancer, Breast Cancer	NCT00949559<br>NCT01485848	1<br>2	KFAKFAKKFAKFAKKFAKQHWSYG-Lau-RPCONHBn	27(1)	A	IV
**Balixafortide**[a] (POL6326)	Polyphor Ltd.	Cxc Chemochine Receptor 4	Metastatic breast cancer	NCT03786094<br>NCT01413568	3<br>1/2	AC(1)SAP-Dab-RYC(1)YQKPPYH	16(1)	H	Oral
**BNZ132-1-40** (BNZ-1)	Bioniz Therapeutics	IL-2, IL-9, IL-15 Cytokines	Cancer and autoimmune diseases	NCT03239392	1/2	IKEFLQRFIHIVQSIINTS	19	H	IV
**ALM 201** (ALM201/0001; ALM-201)	Almac Discovery	Surface Receptor CD44	Solid Tumor, Ovarian Cancer	NCT03427073	1	IRQQPQDPPTETLELEVSPDPAS	23	H	SC
**Tyroserleutide** (YSL)	Shenzedn Kangzhe Pharmaceutical Co.	Protein Expression Of Cam;Activity of PI3K	Primary Hepatocellular Carcinoma	NCT03516448	3	YSL	3	U	Oral

Legenda: Nal: 2-naphtyl-alanine; Pal: palmitic acid; hCit: homocitrulline; Nle: norleucine; Lau: aminolauric acid; Dab: 2,4-diaminobutyric acid
[a]Balixafortide is also active on another pathology: see Table 5. [b]A=Analogous; N=Native; H=Heterologous; U=Undisclosed.

Table 2S: Peptides with protein-protein interaction against uncontrolled inflammation and autoimmune response under clinical trial.

Name (alternative)	Company	Biological Target	Target Disease	Clinical Trial Identifier	Clinical Phase	Structure[a]	AA (UAA)	Class[a]	RoA	RoA
**Forigerimod** (Lupuzor™; IPP-201101)	ImmuPharma	T-cells	Systemic Lupus Erythematosus (SLE)	NCT03427151	3	RIHMVYSKRO(p)SGKPRGYA FIEY	21 (1)	H	SC	SC
**Difelikefalin** (CR 845, KORSUVA®)	Cara Therapeutics	peripheral k-opioid receptor	Pruritus, postoperative pain	NCT03636269<br>NCT03995212<br>NCT04706975<br>NCT02542384	NDA<br>2<br>2<br>2/3	fflk-[γ-(4-N-piperidinyl)amino carboxylic acid]	5 (5)	H	IV<br>Oral<br>Oral<br>IV	IV<br>Oral<br>Oral<br>IV
**EA 230** (Peptide 46)	Exponential Biotherapies	hormonal milieu	Systemic Inflammatory Response Syndrome (SIRS)	NCT03145220	2	AQGV	4	H	IV	IV
**LSALT peptide** (Metablok™)	Arch Biopartners	DPEP-1	Acute Respiratory Distress Syndrome (ARDS)	NCT04402957	2	NH3-LSALTPSPSWLKYKAL-COOH	16	H	IV	IV
**Brimapitide** (AM-111, XG-102, Sonsuvi®)	Auris Medical	Mitogen-activated protein kinase 8	Acute Sensorineural Hearing Loss (ASNHL) and ocular inflammation	NCT02508337	3	dqsrpvqpflnlttprkprpprrrqrrkkrG	31 (30)	H	S-CONJUNC	SC
**Timbetasin** (RGN259, Thymosin B4, Thymosin beta-4)	RegeneRX Biopharmaceuticals	Thymosin β(4)	Dry eye	NCT03937882	3	Ac-SDKPDMAEIEKFDKSKLKKTETQEKNPLPSKETIEQEKQAGES	43	A	OPHTHALM	Ophtalm
**TPX-100**	OrthoTrophix	SIBILING proteins	Knee osteoarthritis	NCT02837900	2	U	U	U	I-ARTIC	I-Artic
**Aviptadil** (RLF-100™, ZYESAMI™)	NeuroRX and Relief Therapeutics Holding	VPAC1 on AT2 cells	Pulmonary Hypertension (PH) and Acute Respiratory Distress Syndrome (ARDS)	NCT04360096	3	HSDAVFTDNYTRLRKQMAV KKYLNSILN	28	H	NASAL	Respir
**Larazotide** (AT-1001; INN-202; Larazotide acetate; SPD 550)	9 Meters Biopharma	Zonulin Receptor	Celiac disease	NCT03569007	3	GGVLVQPG	8	A	Oral	Oral

Legenda: (p)S: serine phosphate. [a]A=Analog; N=Native; H=Heterologous; U=Undisclosed.

Table 3S: Peptides with protein-protein interaction against hormonal hearing and visual genetic diseases under clinical trial.

Name (alternative)	Company	Biological Target	Target Disease	Clinical Trial Identifier	Clinical Phase	Structure	AA (UAA)[a]	Class[a]	RoA
**HTL0030310**	Sosei Heptares	Somatostatin receptor 5	Endocrine disorders	NCT03847207	1	U	U	U	SC
**Vosoritide** (BMN-111)	BioMarin Pharmaceutical	Fibroblast growth factor receptor 3; Atrial natriuretic factor receptor B	Achnondroplasia	NCT03424018<br>NCT04219007	NDA<br>2	PGQEHPNARKYKGANKKGLSK GC(1)FGLKLDRIGSMSGLGC(1)	39	H	SC

[a]A=Analog; N=Native; H=Heterologous; U=Undisclosed.

Table 4S: Peptides with protein-protein interaction designed from proglucagone fragments against diabetes, obesity, SBS and hyperinsulinemia under clinical trial.

Name	Company	Biological Target	Target Disease	Clinical Trial Identifier	Clinical Phase	Structure[a]	AA(UAA)	CLASS[a]	RoA
**G3215**	Imperial College	GLP-1R	Type 2 Diabetes Mellitus/Obesity	NCT02692040	1	U	U	A	SC
**Noiiglutide** (HTI-2088; HS20004)	Jiangsu Hansoh Pharmaceutical	GLP-1R	Type 2 Diabetes Mellitus	NCT03848793	2	H-Aib-EGTFTSDVSSYLEEEAAKEFIAWLVRGGPSSGA PPPSK-Pal	40(1)	A	SC
**PB-119** (PEG Exenatide)	PegBio Co. Ltd	GLP-1R	Type 2 Diabetes Mellitus	NCT04504370; NCT04504396	3	HGEGTFTSDLSKQMEEEAVRLFIEWLKNGGPSS GAPPPC(PEG)-NH_2	39	A	SC
**Avexitide** (exendin 9-39)	Eiger BioPharmaceuticals	GLP-1R	Hyperinsulinemic Hypoglycemia	NCT04652479	2	DLSKQMEEEAVRLFIEWLKNGGPSSGAPPPS-NH_2	31	A	SC
**Apraglutide** (FE203799)	VectivBio	GLP-2R	Short Bowel Syndrome	NCT04627025	3	HGDGSFSDE-Nle-fTILDLLAARDFINWLIQTKITD-NH_2	33(2)	A	SC
**Glepaglutide** (ZP1848)	Zealand Pharma	GLP-2R	Short Bowel Syndrome	NCT03690206	3	HGEGTFSSELATILDALAARDFIAWLIATKITDK KKKKK-NH_2	39	A	SC
**Dapiglutide** (ZP7570)	Zealand Pharma	GLP-1R/ GLP-2R	Short Bowel Syndrome	NCT04612517	1	U	U	U	SC
**CT-868**	Carmot Therapeutics	GLP-1R/ GIPR	Type 2 Diabetes Mellitus	ACTRN126180 01988246	1	U	U	U	SC
**SCO-094** (TAK-094)	Scohia Pharma	GLP-1R/ GIPR	Type 2 Diabetes Mellitus	JapicCTI-205323	1	U	U	U	SC
**Tirzepatide** (LY3298176)	Eli Lilly	GLP-1R/ GIPR	Type 2 Diabetes Mellitus	NCT03861039	3	Y-Aib-EGTFTSDYSI-Aib-LDKIAQK((AEEA)$_2$-γ-E-C20-diacid)-AFVQWLIAGGPSSGAPPPS -NH_2	39(2)	A	SC
**Pegapamodutide** (LY2944876; TT401; MOD-601; OPK88003)	OPKO Health	GLP-1R/ GCGR	Type 2 Diabetes Mellitus	NCT03406377	2	H-Aib-QGTFTSDYSKYLDSKKAQEFVQWLLN-Aib-GRNRNNIAC(MAL-PEG)-C(MAL-PEG)-NH_2	39(2)	A	SC

**BI456906** (ZP2929)	Zealand Pharma	GLP-1R/ GCGR	Type 2 Diabetes Mellitus/Obesity/ NASH	NCT04771273	2	U	U	U	SC
**Cotadutide** (MEDI0382)	Astra Zeneca	GLP-1R/ GCGR	Type 2 Diabetes Mellitus/Obesity	NCT03555994	2	HSQGTFTSDK(γ-E-Pal)-SEYLDSERARDFVAWLEAGG	30	A	SC
**ALT-801** (SP1373; VPD-107)	AltImmune	GLP-1R/ GCGR	NASH	NCT04561245	1	H-Aib-QGTFTSDYSKYLDE*K(GC18c)-AAK*EFIQWLLQT-NH_2	29(1)	A	SC
**AM833** (NN9838; NNC0174-0833)	Novo Nordisk	AMY	Obesity	NCT03856047	2	U	U	A	SC
**LY3305677 (OXM3, IBI362)**	Ely Lilly	GLP-1R/ GCGR	Obesity/Diabetes	NCT03928379	2	U	U	A	SC
**PYY-1875** (NNC0165-1875)	Novo Nordisk	Y2R	Obesity	NCT03707990	1	U	U	A	SC
**GT-001** (PeptideYY(3-36))	Gila Therapeutics	Y2R	Obesity/Diabetes	NCT03490786	1	IKPEAPGEDASPEELNRYYASLRHYLNLVTRQRY-NH_2	34	N	TOPIC
**LY3437943**	Ely Lilly	GLP-1R/ GIPR/ GCGR	Obesity/Diabetes	NCT04143802	1	U	U	A	SC
**Dasiglucagon** (ZP 4207; HypoPal®)	Zealand Pharma	GCGR	Hyperinsulinemic Hypoglycemia/		NDA	HSQGTFTSDYSKYLD-Aib-ARAEEFVKWLEST	29(1)	A	SC

Legenda: Aib: 2-aminoisobutyric acid; Pal: palmitic acid; Nle: norleucine; PEG: polyethylene glycol; AEEA: 8-amino-3,6-dioxaoctanoic acid dimer; C20-diacid: eicosanedioic acid; Mal: maleimide.
[a]A=Analogous; N=Native; H=Heterologous; U=Undisclosed. [b] See text for detailed description of the structure.

Table 5S: Peptides as drug for the treatment of cardiovascular system and hypertension with protein-protein interaction under clinical trial.

Name	Company	Biological Target	Target Disease	Clinical Trial Identifier	Clinical Phase	Structure	AA (UAA)	Class[b]	RoA
**Ularitide** (Urodilatin)	Cardiorentis	Natriuretic peptide receptor-A	Acute Decompensate Heart Failure (ADHF)	NCT01661634	3	YAPRSLRRSSC(1)FGGRMDRIGAQSGLGC(1)NSFRY	32	A	IV
**CN-105** (Ac-VSRRR-NH2)	AegisCN	LDL receptor	Intracerebral Hemorrhage/Stroke	NCT03168581	2	Ac-VSRRR-NH_2	5	A	IV
**Pemziviptadil** (PB1046, Vasomera™)	PhaseBio Pharmaceuticals	Vasoactive intestinal peptide type II receptor	Pulmonary Arterial Hypertension	NCT03556020	2	ELP-MHSDAVFTDNYTRLRKQMAVKKYLNSILN	29	A	SC
**Nerinetide** (NA-1, Tat-NR2B9c)	NoNO Inc.	PSD-95	Stroke, Acute	NCT04462536	3	YGRKKRRQRRRKLSSIESDVYGRKKRRQRRR	31	A	IV
**Balixafortide[a]** (POL6326)	Polyphor Ltd.	Cxc Chemochine Receptor 4	Large Reperfused ST-Elevation Myocardial Infarction	NCT01905475	2	AC(1)SAP{Dab}RYC(1)YQKPPYH	16	H	Oral

Legenda: Dab: 2,4-diaminobutyric acid;
[a]Balixafortide is also active on other pathologies: see Table 1. [b]A=Analogous; N=Native; H=Heterologous; U=Undisclosed

Table S6: Peptides as drug for the treatment of degenerative diseases with protein-protein interaction under clinical trial.

Name	Company	Biological Target	Target Disease	Clinical Trial Identifier	Clinical Phase	Structure	AA (UAA)	Class[a]	RoA
**Alirinetide** (GM6-GM604)	Genervon	Multi-target	Amniotropic lateral sclerosis	NCT01854294	2	FSRYAY	6	N	IV
**Davunetide** (NAP, AL-108)	Allon Therapeutics/University of California, San Francisco (UCSF)	Microtubule-associated protein modulators;	Progressive supranuclear palsy	NCT01056965	1	NAPVSIPQ	8	N	NASAL
**RA101495** (Zilucoplan)	UCB Pharma	Complement C5 inhibitors	Myasthenia gravis	NCT04225871	3	Ac-K(1)-VERFD(1)-D(NMe)-G(tBu)-YW(Aza)-EYPG(Cy)-K-PEG- γ-E-Pal	15(4)	H	SC
**NLY01** (TLY 001)	Neuraly	GLP-1R	Alzheimer/ Parkinson Disease	NCT04154072	2	HGEGTFTSDLSKQMEEEAVRLFIEWLKNGGPSSGAPPPSC-PEG	40	A	SC

Legenda: W(Aza): 7-azatryptophan, G(Cy): N-cyclohexyl-glycine; Pal: palmitic acid; PEG: polyethylene glycol
[a]A=Analogous; N=Native; H=Heterologous.

Table S7: Peptides as antibiotic and antiviral with protein-protein interaction under clinical trial.

Name	Company	Biological Target	Target Disease	Clinical Trial Identifier	Clinical Phase	Structure	AA (UAA)	Class[a]	RoA
**Nangibotide** (Motrem™, LR-12)	Inotrem	TREML1 protein inhibitors	Shock, Septic; COVID 2019 infections	NCT04055909<br>NCT04429334	2<br>2	LQEEDAGEYGCM-NH_2	12	N	IV
**Reltecimod** (AB103)	Atox Bio	B7-2/CD28	necrotizing soft tissue infection.	NCT02469857	NDA	aSPMLVAYDa	10 (2)	A	IV
**C16G2**	armata pharm.	Mechanism unclear	Dental Caries	NCT03196219	2	TFFRLFNRSFTQALGKGGGKNLRIIRKGIHIIKKY-NH_2	35	A	Oral

[a]A=Analog; N=Native; H=Heterologous.

Table S8: Peptides with protein-protein interaction against different pathologies under clinical trial.

Name	Company	Biological Target	Target Disease	Clinical Trial Identifier	Clinical Phase	Structure	AA (UAA)	Class[a]	RoA
**Hepcidin** (LJPC-401)	La Jolla Pharm.Com.	Ferroportin-1	Hereditary Hemochromatosis	NCT03395704	2	DTHFPIC(1)IFC(2)C(3)GC(2)C(4)HRSKC(3)GMC(4)C(1)KT	**25**	N	SC
**Ghrelin** (OXE-103, SUN11031)	Oxeia Biopharm.	Unclear	Concussion, Brain Traumatic Brain Injury	NCT04558346	2	GSS(octadecyl)FLSPEHQRVQQRKESKKPPAKLQPR	28	N	SC
**Solnatide** (AP-301)	Apeptico	ENaC	Acute lung injury; Adult respiratory distress syndrome.	NCT03567577	2	C(1)GQRETPEGAEAKPWYC(1)	17	A	NASAL
**TAK639** (SHP 639)	Takeda	type B natriuretic peptide receptor	Ocular hypertension/primary open-angle glaucoma (POAG)	NCT03131167	1	(S)-N-octanoyl-nipecotic acid-F-(R)-(N′, N′) dimethylornithine-L-(3-hydroxy)PLDRI-NH_2	9	H	OPHTHALM

[a]A=Analog; N=Native; H=Heterologous.
